# Progress toward a Better BOCS: Systematic Coarse-Graining with Local Density Potentials

**DOI:** 10.1021/acs.jctc.6c01228

**Published:** 2026-10-12

**Authors:** Maria C. Lesniewski, Michael R. DeLyser, W. G. Noid

**Affiliations:** Department of Chemistry, 8082 The Pennsylvania State University, University Park, Pennsylvania 16802, United States

## Abstract

We introduce version 5.0 of the bottom-up open-source coarse-graining software (BOCS) package. BOCS employs the force-matching variational principle to parametrize potentials for coarse-grained (CG) models directly from atomically detailed simulations. BOCS version 5.0 significantly extends previous versions by treating potentials that depend upon the local density (LD) around each particle as well as potentials that depend upon the square gradient (SG) of this local density. We also describe a new package, PKG-BOCS, for simulating these potentials with LAMMPS. This software treats complex molecular topologies and provides considerable flexibility for defining the local density as well as the LD and SG potentials. We present numerical calculations that provide physical insight into these potentials and their dependence on the definition of the local density. These calculations also demonstrate the accuracy and reliability of our implementation. Finally, we demonstrate that LD potentials can improve the structural fidelity, thermodynamic properties, and transferability of CG models for water.

## Graphical abstract

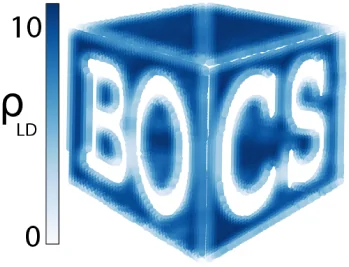

## 1 Introduction

By representing systems in reduced detail, coarse-grained (CG) models provide the necessary computational efficiency for simulating length- and time-scales that cannot be easily addressed with atomically detailed models.^1–4^ Coarse-graining also provides conceptual advantages by eliminating unnecessary details and focusing attention on essential features.^5–7^ There exist many approaches for developing CG models with varying advantages and limitations.^8–10^ Here, we focus on “bottom-up” CG models that are parameterized with information from simulations of all-atom (AA) or other high-resolution models.^11,12^ In principle, statistical mechanics provides a framework for parameterizing bottom-up models that exactly reproduce all structural and thermodynamic properties of the underlying AA model that can be observed at the resolution of the CG model.^13,14^ In practice, though, this exact coarse-graining procedure is not analytically tractable and must be computationally approximated.

Several open-source software packages implement bottom-up methods for approximating this exact coarse-graining procedure, including the versatile object-oriented toolkit for coarse-graining applications (VOTCA),^15,16^ IBIsCO,^17^ MagiC,^18,19^ the bottom-up open-source coarse-graining software (BOCS) package,^20^ and the Open multiscale coarse-graining^21^ (OpenMSCG) package.^22,23^ VOTCA, IBIsCO, MagiC, and OpenMSCG implement structure-based methods that iteratively refine CG potentials to reproduce target distribution functions describing the mapped ensemble, i.e., the ensemble obtained by mapping the equilibrium ensemble for the underlying AA model to the CG representation.^24–26^ If they converge, these methods maximize the likelihood that the CG model will reproduce the mapped ensemble.^27–29^ Conversely, VOTCA, BOCS, and OpenMSCG implement a force-matching^30–33^ variational principle^21,34^ that directly (i.e., noniteratively) parametrizes the CG potential to optimally approximate the configuration-dependence of the exact CG potential when evaluated over the mapped ensemble. This force-matching variational approach is closely related^35^ to the score-matching method^36^ for parametrizing machine learning methods.^37^

BOCS complements other force-matching implementations by providing several unique capabilities. For instance, BOCS employs a generalized Yvon–Born–Green (g-YBG) framework^38,39^ to perform force matching without forces by directly determining the CG potential from structural information.^40,41^ This g-YBG framework clarifies and quantifies the relationship between the mapped ensemble and the CG potential.^39,42^ Additionally, BOCS implements a simple framework for improving the transferability of CG potentials across multiple systems and thermodynamic conditions.^43^ Moreover, BOCS includes software for parametrizing configuration-independent potentials of the volume.^44,45^ CG models with these volume potentials can quantitatively reproduce the AA pressure–density equation of state across a very wide range of thermodynamic conditions.^46,47^ Volume potentials can also reasonably describe density fluctuations in metal-organic frameworks.^48^ However, volume potentials cannot be employed to model liquid-vapor interfaces or other highly inhomogeneous systems.

A growing number of studies have modeled such inhomogeneous systems by supplementing conventional pair potentials with potentials of the local density around each molecule. By defining the local density with additive contributions from surrounding molecules, local density (LD) potentials generate short-range pair forces that are sensitive to the local environment. While conceptually similar to embedded atom models for metals,^49,50^ LD potentials for soft materials were first introduced in the many-body dissipative particle dynamics model developed by Pagonabarraga and Frenkel.^51^ Wolynes and coworkers independently introduced LD potentials for modeling water-mediated interactions in protein structure prediction.^52^ More recently, bottom-up models have employed LD potentials to describe liquids,^53–60^ liquid mixtures,^61–63^ polymers,^64–67^ and even explosive materials.^68,69^ These LD potentials can significantly improve the pressure–density equation of state for bottom-up models^55,56^ and provide outstanding transferability between bulk and interfacial environments.^57^ LD potentials also reasonably reproduce the cooperative many-body interactions that govern hydrophobic phenomena.^70^ Moreover, following the analogy to classical density functional theory,^51,71^ we have introduced a new class of interaction potentials that depend upon both the local density and the square of its gradient.^72^ The combination of these LD and square gradient (SG) potentials can model interfacial density profiles very accurately while still employing only pair-additive forces.

Here, we describe a new release of BOCS for parametrizing LD and SG potentials via variational force matching. We also describe a new version of the PKG-BOCS package for simulating these potentials in the LAMMPS molecular dynamics engine.^73,74^ This software allows for multiple types of particles and corresponding local densities.^59^ It allows users to define each type of local density with a unique length scale and with one of five different weighting functions. The software implements simple functional forms and also more flexible spline-based representations for the LD and SG potentials. Additionally, this software is compatible with complex CG topologies while properly accounting for intramolecular contributions and exclusions. The updated package includes detailed documentation and tutorials that describe and illustrate the software. Importantly, the present calculations validate the accuracy and reliability of this software by quantitatively determining tens of thousands of parameters for a condensed-phase system with 125 molecules that are governed by bond, angle, dihedral, pair, LD, and SG potentials. We hope that this new software will provide a useful complement to existing packages for parametrizing and simulating LD potentials.^23,62,64^

The remainder of this paper is organized as follows: the theory section describes the relevant background for LD and SG potentials. The methods section describes the computational workflow for parametrizing and simulating these potentials. This section also summarizes the details of the numerical calculations that illustrate this new software. The results section presents numerical calculations that illustrate the accuracy and reliability of the new software while providing physical insight into LD and SG potentials. The concluding section provides closing comments and suggests promising directions for future work. The appendix provides further analysis of LD and SG potentials, while the Supporting information (SI) provides additional computational details.

## 2 Theory

### 2.1 Mapped Ensemble

Bottom-up approaches parameterize CG models based upon information from a higher-resolution model.^11,12^ We shall refer to this high-resolution model as an all-atom (AA) model for simplicity, but the present framework applies more generally to classical particle-based models. We denote the configuration of the AA model by **r** = (**r**_1_, ..., **r**_*n*_), where **r**_*i*_ denotes the Cartesian coordinates of atom *i*. We assume that the system is at equilibrium such that the AA model samples each configuration according to the canonical probability density, *p*_r_(**r**) ∝ exp­[−β*u*(**r**)], where β = 1/*k*_*B*_ *T* is the inverse temperature and *u*(**r**) is the corresponding potential.^75^ While we focus on the canonical ensemble, this framework can be generalized to other ensembles.^44,45^

We employ a mapping, **M** : **r** → **R** = **M**(**r**), to define the CG representation, **R** = (**R**_1_, ..., **R**_*N*_), of each AA configuration.^21^ This mapping defines the coordinates of each site, *I*, as a convex combination of atomic coordinates, i.e., $R_{I}=\overset{n}{\underset{i=1}{\sum }}c_{Ii}r_{i}$ .^76^ In particular, we consider “non-overlapping” maps for which each atom contributes to the definition of at most one CG site. We define a restricted partition function for the total Boltzmann weight associated with each CG configuration, **R**:

1 $$z_{R}\left(R\right)\equiv \int_{D_{n}\left(V\right)}dr\;\exp\left[-\beta u\left(r\right)\right]\delta \left(M\left(r\right)-R\right)$$

where $D_{n}\left(V\right)$ denotes the set of AA configurations that are accessible when the system has volume *V*.^76^ This restricted partition function determines the mapped probability density, *p*_R_(**R**) ∝ *z*_R_(**R**), as well as the exact CG potential:

2 $$W\left(R\right)\equiv -k_{B}T\;\ln\;[z_{R}\left(R\right)/V_{0}^{n-N}]$$

where *V*_0_ is a constant reference volume.^21^ If *W* is known as a function of both configuration and also thermodynamic conditions, then it is possible to quantitatively reproduce all structural and thermodynamic properties that are observable at the resolution of the CG mapping.^13,14^

### 2.2 Approximate CG Potential

BOCS approximates *W* with CG potentials of the form

3 $$U\left(R,V\right)\equiv U_{b}\left(R\right)+U_{nb}\left(R\right)+U_{V}\left(V\right)$$

Here, *U*_b_(**R**) is a conventional bonded potential, *U*_nb_(**R**) is the nonbonded potential, and *U*_V_(*V*) is a volume potential.

The bonded and volume potentials in eq 3 have not changed in this latest version of BOCS. The bonded potential includes terms governing bond, angle, and torsional degrees of freedom. These terms can be defined with simple analytic forms (e.g., harmonic bond and angle potentials) or with more flexible basis functions (e.g., linear or cubic spline functions). The volume potential does not generate forces on any particles but directly contributes to the internal pressure via *F*_V_(*V*) = −d*U*_V_(*V*)/d*V*. BOCS can represent this volume force with a two parameter functional form^44^ or with more flexible basis functions.^45–47^

BOCS now treats three different classes of non-bonded interactions:

4 $$U_{nb}\left(R\right)\equiv U_{pair}\left(R\right)+U_{LD}\left(R\right)+U_{SG}\left(R\right)$$

Here, *U*_pair_, *U*_LD_, and *U*_SG_ denote, respectively, contributions from conventional central pair potentials, local density potentials, and also potentials that depend upon the square of the local density gradient. BOCS allows for complex molecular topologies and many different types of sites. However, for the sake of simplicity and clarity of presentation, we shall assume that all CG particles are of the same type in the present subsection.

For each CG site, *I*, we define a set, $S_{I}$ , of sites that interact with *I* via non-bonded potentials. This set can include intramolecular non-bonded interactions while also accounting for intramolecular exclusions. The pair contribution to *U*_nb_ may be expressed

5 $$U_{pair}\left(R\right)\equiv \frac{1}{2}\overset{N}{\underset{I=1}{\sum }}\underset{J\in S_{I}}{\sum }U_{2}\left(R_{IJ}\right)$$

where *U*_2_ is a central pair potential and *R*_*IJ*_ is the distance between CG sites *I* and *J*.

Previous releases of BOCS did not treat *U*_LD_ and *U*_SG_. These terms both depend upon the instantaneous local density, ρ_*I*_, around each particle, *I*. We define ρ_*I*_ as a sum of contributions from neighboring particles that participate in nonbonded interactions with *I*:

6 $$\rho_{I}\equiv \rho_{I}\left(R\right)\equiv c+\underset{J\in S_{I}}{\sum }\mathrm{w̅}\left(R_{IJ}\right)$$

Here, *w̅*(*r*) is a normalized, nonincreasing weighting function that vanishes for all *r* ≥ *r*_c_, while *c* is a configuration-independent constant that can be ignored, *c* = 0, or used to define the “self-contribution,” *c* = *w̅*(0). BOCS allows users to specify the LD length-scale, *r*_c_, and to employ five different functional forms for *w̅*. We discuss these weighting functions in Section 3.1.3 below and Section I of the SI. We now define

7 $$U_{LD}\left(R\right)\equiv \overset{N}{\underset{I=1}{\sum }}U_{\rho }\left(\rho_{I}\right)$$

8 $$U_{SG}\left(R\right)\equiv \overset{N}{\underset{I=1}{\sum }}U_{\nabla }\left(\rho_{I}\right){|\nabla_{I}\rho_{I}|}^{2}$$

where **∇**_*I*_ρ_*I*_ = ∂ρ_*I*_/∂**R**_*I*_. Note that *U*_**∇**_ is not a potential and does not have units of energy. Rather, *U*_**∇**_ is a coefficient that modulates the square gradient contribution as a function of the local density and, in particular, can be defined to act primarily in interfacial regimes.^72^ Because they are defined in terms of the local density, eq 6 ensures that *U*_LD_ and *U*_SG_ properly treat intramolecular contributions and exclusions.

The total non-bonded force on site *I* may be expressed as

9 $$F_{I;nb}\equiv -\nabla_{I}U_{nb}\left(R\right)=\underset{J\in S_{I}}{\sum }F_{IJ;nb}\left(R\right)$$

where the total non-bonded force between the pair (*I*, *J*) is

10 $$F_{IJ;nb}\left(R\right)\equiv \left\{F_{2}\left(R_{IJ}\right)+\left[F_{\rho }\left(\rho_{I}\right)+F_{\rho }\left(\rho_{J}\right)\right]{\mathrm{w̅}}^{\prime }\left(R_{IJ}\right)\right\}{\mathrm{R̂}}_{IJ}+F_{IJ;SG}\left(R\right)$$

Here, *F*_2_(*r*) = −d*U*_2_(*r*)/d*r*, *F*_ρ_(ρ) = −d*U*_ρ_(ρ)/dρ, *w̅*′(*r*) = d*w̅*(*r*)/d*r*, **R̂**_*IJ*_ is the unit vector pointing from *J* to *I*, and **F**_*IJ*;SG_(**R**) is the contribution to the pair force from *U*_SG_. Because eq 6 defines the local density as a function of pair distances, both *U*_pair_ and *U*_LD_ generate antisymmetric forces along the vector **R̂**_*IJ*_ between each pair. Thus, *U*_LD_ modulates the net force between the pair in eq 10 to reflect the local densities, ρ_*I*_ and ρ_*J*_, of the pair (*I*, *J*). Moreover, this implies an important relationship between *U*_2_ and *U*_ρ_. Specifically, for any constant *c*, the transformation of the corresponding force functions

11 $$\begin{array}{lll} F_{2}\left(r\right)\to {\mathrm{F̃}}_{2}\left(r;c\right) & \equiv & F_{2}\left(r\right)+c{\mathrm{w̅}}^{\prime }\left(r\right)\\ F_{\rho }\left(\rho \right)\to {\mathrm{F̃}}_{\rho }\left(\rho ;c\right) & \equiv & F_{\rho }\left(\rho \right)-c/2 \end{array}$$

leaves the net pair force, **F**_*IJ*;nb_, unchanged.^56^ Consequently, there exists a non-trivial, one-parameter family of pair and LD potentials that generate identical forces and, therefore, identical equilibrium distributions.

The contribution from *U*_SG_ may be expressed as^72^

12 $$\begin{array}{ll} F_{IJ;SG}\left(R\right)\equiv & \left[F_{\nabla }\left(\rho_{I}\right){|\nabla_{I}\rho_{I}|}^{2}+F_{\nabla }\left(\rho_{J}\right){|\nabla_{J}\rho_{J}|}^{2}\right]{\mathrm{w̅}}^{\prime }\left(R_{IJ}\right){\mathrm{R̂}}_{IJ}\\ & -2g\left(R_{IJ}\right)\left[A_{IJ}\cdot {\mathrm{R̂}}_{IJ}\right]{\mathrm{R̂}}_{IJ}-2h\left(R_{IJ}\right)A_{IJ} \end{array}$$

where *F*_**∇**_(ρ) = −d*U*_**∇**_(ρ)/dρ, *h*(*r*) ≡ *w̅*′(*r*)/*r*, *g*(*r*) = *w̅*″(*r*) – *h*(*r*), and

13 $$A_{IJ}=U_{\nabla }\left(\rho_{I}\right)\nabla_{I}\rho_{I}-U_{\nabla }\left(\rho_{J}\right)\nabla_{J}\rho_{J}$$

While the SG potential generates antisymmetric pair-additive forces, these forces are not along **R̂**_*IJ*_ because the last term in eq 12 introduces contributions along **∇**_*I*_ρ_*I*_ and **∇**_*J*_ρ_*J*_. Because *w̅*(*r*) = 0 for *r* > *r*_c_, the LD and SG contributions to **F**_*IJ*;nb_(**R**) also vanish when *R*_*IJ*_ > *r*_c_.

Equations 5–13 explicitly consider a single type of particle and, consequently, a single local density. Appendix 1 generalizes this treatment for CG models with many different types of particles and local densities, along with a corresponding diversity of LD and SG potentials.

### 2.3 Variational Force-Matching

BOCS determines the optimal approximate CG potential according to the force-matching variational principle^21,31^ that is employed in the multiscale coarse-graining (MS-CG) method,^32,33^ i.e., by minimizing

14 $$\chi^{2}\left[U\right]\equiv \left\langle \frac{1}{3N}\overset{N}{\underset{I=1}{\sum }}{|F_{I}(M(r))-f_{I}(r)|}^{2}\right\rangle$$

Here, the angular brackets denote an average over the equilibrium ensemble for the AA model, **f**_*I*_(**r**) indicates the net force on site *I* in the AA configuration **r**,^21,77^ and **F**_*I*_(**R**) = –**∇**_*I*_ *U*(**R**); eq 14 can be decomposed^21^ into a fixed contribution from the exact CG potential, *W*, and a non-negative contribution comparing the gradients of *W* and *U*:

15 $$\chi^{2}\left[U\right]=\chi^{2}\left[W\right]+\int_{D_{N}\left(V\right)}p_{R}\left(R\right)\frac{1}{3N}\overset{N}{\underset{I=1}{\sum }}{|\nabla_{I}\left(W-U\right)|}^{2}$$

Consequently, *W* provides the global minimum of χ^2^[*U*]. Moreover, variations in the CG potential, *U* → *U* + δ*U*, that reduce χ^2^ result in a better approximation for the configuration-dependence of *W*. We now construct a basis set for performing this variational calculation.^34^

Equations 9 and 10 demonstrate that the total nonbonded force, **F**_*I*;nb_, on each particle, *I*, depends linearly upon the force functions, *F*_2_ and *F*_ρ_, that are determined by the pair and LD potentials, *U*_2_ and *U*_ρ_. Similarly, the total bonded force from *U*_b_ in eq 3 may also be written as a linear combination of bonded force functions. For each bonded, pair, or LD potential, *U*_ζ_(*x*), we represent the corresponding force function, *F*_ζ_(*x*) = −d*U*_ζ_(*x*)/d*x*, by a linear combination of basis functions, *f*_ζ*d*_(*x*), with constant coefficients, ϕ_ζ*d*_:

16 $$F_{\zeta }\left(x\right)=\underset{d}{\sum }\phi_{\zeta d}f_{\zeta d}\left(x\right)$$

This basis expansion allows us to express the CG force, **F**_*I*_, on each particle, *I*, as a linear combination of these coefficients, ϕ_ζ*d*_. However, this approach does not work for the SG potential because the corresponding force in eq 12 depends upon both *F*_**∇**_ and *U*_**∇**_. Consequently, we represent *U*_**∇**_(*x*) = ∑_*d*_ ϕ_**∇** *d*_ *f*_**∇** *d*_(*x*), which determines a corresponding representation for $F_{\nabla }\left(x\right)=-\underset{d}{\sum }\phi_{\nabla d}f_{\nabla d}^{\prime }\left(x\right)$ . This allows us to express the total force on each CG site as a linear combination of these coefficients:

17 $$F_{I}\left(R;\phi \right)=\underset{\zeta }{\sum }\underset{d}{\sum }\phi_{\zeta d}G_{I;\zeta d}\left(R\right)$$

where $G_{I;\zeta d}$ specifies the direction of the force on CG site *I* that arises from the *d* ^th^ basis function, *f*_ζ*d*_(*x*), for either the ζ force function, *F*_ζ_(*x*), or, in the case of the SG interaction, ζ = **∇**, the coefficient function, *U*_**∇**_(*x*). The coefficient, ϕ_ζ*d*_, then determines the magnitude of this force. Appendix 1 provides explicit expressions for the vector $G_{I;\zeta d}\left(R\right)$ , associated with each type of interaction.

If we now consider **F**_*I*_(**R**; ϕ) to be the *I* ^th^ element of a force field, **F**(ϕ) = {**F**_*I*_(**R**; ϕ)}, and adopt a “super-index” *D* = ζ*d*, then eq 17 may be re-expressed more simply as

18 $$F\left(\phi \right)=\underset{D}{\sum }\phi_{D}G_{D}$$

where $G_{D}=\left\{G_{I;D}\left(R\right)\right\}$ may be considered a force field basis vector.^34^ The set, $\left\{G_{D}\right\}$ , of basis vectors included in eq 18 specifies a vector space of CG force fields that is defined by the form of the approximate potential, *U*. A set of coefficients, ϕ ≡ {ϕ_*D*_}, identifies a particular force field within this subspace and determines a corresponding approximate potential (to within an irrelevant additive constant). However, if the basis functions, *f*_ζ*d*_(*x*), for the pair and LD force functions in eq 16 are sufficiently flexible to represent the transformation in eq 11, then the basis vectors corresponding to pair and LD forces are not all linearly independent. In this case, there exists a one-parameter family of force field parameters, ϕ­(*c*), that corresponds to the same CG force field.

Given this basis set, χ^2^ becomes a quadratic form in the potential parameters:

19 $$\chi^{2}\left(\phi \right)\equiv \chi^{2}\left[U\left(\phi \right)\right]=\underset{D,D^{\prime }}{\sum }\phi_{D}G_{DD^{\prime }}\phi_{D^{\prime }}-2\underset{D}{\sum }b_{D}\phi_{D}+\chi_{0}^{2}$$

where $\chi_{0}^{2}=\chi^{2}\left[0\right]$ and

20 $$G_{DD^{\prime }}\equiv \left\langle \frac{1}{3N}\overset{N}{\underset{I=1}{\sum }}G_{I;D}\left(M\left(r\right)\right)\cdot G_{I;D^{\prime }}\left(M\left(r\right)\right)\right\rangle$$

21 $$b_{D}\equiv \left\langle \frac{1}{3N}\overset{N}{\underset{I=1}{\sum }}G_{I;D}\left(M\left(r\right)\right)\cdot f_{I}\left(r\right)\right\rangle$$

The set of minimizing coefficients, $\phi_{*}=\left\{\phi_{D_{*}}\right\}$ , that determine the optimal approximate CG potential satisfy the normal system of equations^34^

22 $$\underset{D^{\prime }}{\sum }G_{DD^{\prime }}\phi_{D_{*}^{\prime }}=b_{D}$$

BOCS includes several numerical methods for solving eq 22. Because *G_DD_*_′_ and *b*_*D*_ may be determined from an AA trajectory, the force-matching variational principle directly determines the optimal CG potential without requiring simulations of CG models with trial potentials as in, e.g., iterative Boltzmann inversion^25^ or the inverse Monte Carlo method.^24^

We close this section with several additional comments on variational force-matching. The matrix *G_DD_*_′_ quantifies the correlation between the forces generated by the *D* and *D*′ basis vectors when evaluated over the mapped ensemble.^42^ Similarly, *b*_*D*_ quantifies the correlation in the mapped ensemble between the AA forces and the *D* basis vector.^40^ Because *b*_*D*_ can be related to the AA mean force, it can be computed directly from structural information.^29,41^ This allows BOCS to perform force-matching directly from structural ensembles without force information.^39^ However, while this capability has been implemented in BOCS for bonded, pair, and LD potentials,^20,57^ it has not yet been implemented for SG potentials.

We have found it instructive to define an inner product, ⊙, between force fields based upon their correlation in the equilibrium AA ensemble.^29,35^ Given this definition, $G_{DD^{\prime }}=G_{D}⊙G_{D^{\prime }}$ corresponds to a Gram matrix related to the “angle” between the basis vectors included in the CG force field.^42^ Two basis vectors are only orthogonal if they generate uncorrelated forces. Similarly, $b_{D}=G_{D}⊙f$ is the inner product of the basis vector $G_{D}$ and the AA force field, **f**. The normal system of equations for the CG force field, eq 22, may then be geometrically expressed $G_{D}⊙F=G_{D}⊙f$ . This implies that the approximate CG force field, **F**, and the original AA force field, **f**, have the same projection onto each basis vector, $G_{D}$ . This force-balance relation generalizes the Yvon–Born–Green equation from liquid state theory^78^ to arbitrarily complex potentials.^39,40,57^

## 3 Computational Methods

### 3.1 Modifications to BOCS Version 5.0

#### 3.1.1 Computational Workflow

BOCS implements bottom-up approaches for parameterizing the approximate interaction potential, *U*(**R**, *V*), in eq 3. We have previously described the software for parameterizing the volume potential, *U*_V_(*V*), via self-consistent pressure-matching.^45^ Consequently, we only describe the software for parameterizing the approximate interaction potential, *U*_R_(**R**) ≡ *U*_b_(**R**) + *U*_nb_(**R**).

Figure 1 illustrates the basic workflow for parameterizing *U*_R_(**R**). This workflow begins with an AA trajectory that must include the atomic configuration, **r**_*t*_ = {**r**_*i*_(*t*)}, at one or more times, *t*. The AA trajectory may also include the net force, **f**_*i*_(**r**_*t*_), on each atom, *i*. The cgmap tool reads this AA trajectory file, as well as files that define the CG mapping, **M**, and the topology of the CG model. The mapping file identifies the atoms that are associated with each site and also the corresponding mapping coefficients, *c*_*Ii*_. The cgmap tool is currently compatible with non-overlapping maps that associate the CG sites, *I* = 1, ..., *N*, with disjoint sets, {V_*I*_}_*I*=1,···,*N*_, of 1 or more atoms, i.e., each atom can contribute to 0 or 1 sites. Conversely, the CG topology file specifies the number and types of the sites and molecules in the CG model. This topology file also specifies the composition and bonded connectivity of each molecule type, as well as the excluded intramolecular interactions. The cgmap tool then outputs a CG trajectory file with the mapped representation, **R**_*t*_ = **M**(**r**_*t*_), of each AA configuration, **r**_*t*_. If the AA trajectory file includes force information, then the mapped trajectory file will also include the mapped force, **f**_*I*_(**r**_*t*_), on each CG site, *I*. For the non-overlapping maps that are currently supported, $f_{I}\left(r_{t}\right)=\underset{i\in V_{I}}{\sum }f_{i}\left(r_{t}\right)$ , is simply the net force on the atomic group, V_*I*_, associated with site *I*.^21^ If the approximate CG potential, *U*_R_, does not include LD or SG terms, then cgff can use this mapped trajectory to determine *U*_R._

**Figure 1:**
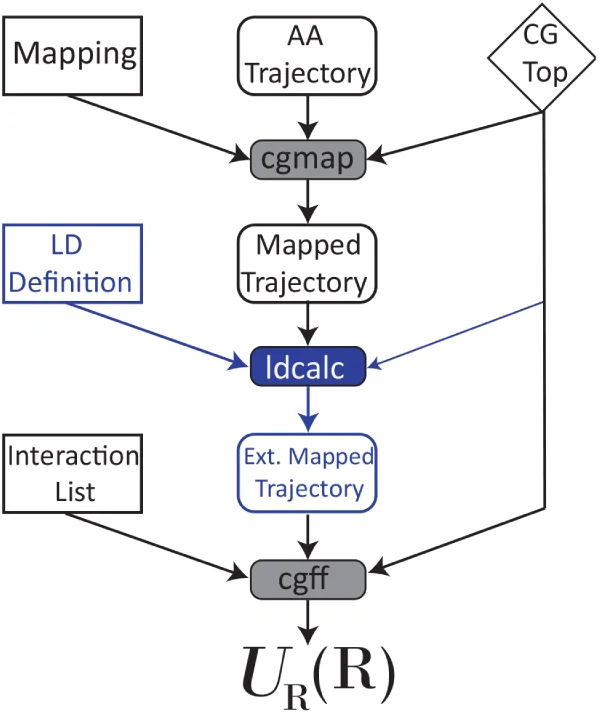
Basic workflow for parameterizing the CG interaction potential, *U*_R_(**R**), with BOCS. Shaded boxes with rounded corners indicate BOCS programs. Unshaded boxes with rounded corners indicate the input AA trajectory and how it is processed through the workflow. Rectangles with sharp corners indicate user-provided input files, while the diamond indicates the CG topology. We highlight in blue the new BOCS components that are required for parameterizing LD or SG potentials.

If *U*_R_ does include LD or SG terms, then we employ the ldcalc tool to supplement the mapped trajectory with the necessary local density information. In addition to the mapped trajectory and the CG topology, ldcalc also reads a file that specifies the weighting function, ${\mathrm{w̅}}_{\tau |\tau^{\prime }}$ , and length-scale, $r_{\tau |\tau^{\prime };c}$ , for the local density associated with each ordered pair, {τ|τ′}, of site types in the CG model. The ldcalc tool then calculates the local density, ρ_τ|*I*_(**R**_*t*_), and the gradient, **∇**_*I*_ρ_τ|*I*_(**R**_*t*_), for each site type, τ, around each CG site, *I*, in each mapped configuration, **R**_*t*_. The ldcalc tool outputs an extended mapped trajectory that includes this additional information.

Finally, the cgff tool determines the coefficients, ϕ_*_, for the interaction potential, *U*_R_(ϕ_*_), that optimally approximates the configuration dependence of the exact potential, *W*. In addition to the (original or extended) mapped trajectory and the CG topology file, the cgff tool reads a file that specifies the various terms in *U*_R_, as well as the options and hyperparameters employed in the variational force-matching calculation. The cgff tool calculates the correlation matrix, *G_DD_*_′_, and the force vector, *b*_*D*_, that are defined by eqs 20 and 21. The cgff tool then solves the normal equations, eq 22 for the optimal coefficients, ϕ_*_. BOCS provides additional Python scripts and utilities that format *U*_R_ for simulations in GROMACS or LAMMPS. However, it appears that versions of Gromacs after 2019 have not supported nonbonded potentials defined by flexible spline basis functions. Moreover, CG potentials with LD or SG terms must be simulated with LAMMPS.

We have worked with LAMMPS developers to extend PKG-BOCS for simulating LD and SG potentials. This extension was released in the 4 July 2026 distribution of LAMMPS. PKG-BOCS can also be used as an analysis tool for calculating the local densities and their gradients around each particle in simulations that do not employ LD or SG potentials. The key new component in PKG-BOCS is a pair_style (ldd) that enables users to combine LD and SG potentials with other pair styles. Our original implementation, which was used for the present calculations and will be hosted on our GitHub page,^79^ also introduced a new ldd atom style. However, the ldd atom_style was refactored into the ldd pair_style in the main-line LAMMPS distribution. We have contributed documentation, unit tests, and examples for PKG-BOCS to the LAMMPS distribution. We are distributing documentation on the Noid group GitHub page for parametrizing LD and SG potentials with BOCS version 5.0,^80^ as well as tutorials for simulating LD potentials with PKG-BOCS.

#### 3.1.2 Software Implementation and Ecosystem

The cgff tool provides an efficient C implementation of force-matching that scales well to large systems and long trajectories. While our original implementation calculated the many-body contributions to *G_DD_*_′_ via a time-consuming triple loop over particles, the current version of cgff calculates *G_DD′_* much more efficiently by directly employing the force field basis vectors. This new implementation only involves a simple pair loop, which should scale analogously to calculating the radial distribution function. Moreover, cgff employs a simple OpenMPI implementation that further reduces the computational time for computing *G_DD_*_′_ and *b*_*D*_ by a factor of n by distributing the sampled configurations over n independent processors.

Figure 2 demonstrates the performance of the cgff calculation for parameterizing the one-site LD-BULK water model that is presented in Section 4.2. Specifically, we employed AMD EPYC 7763 processors with InfiniBand HDR100 interconnections on the Anvil cluster.^81^ Each calculation treated a mapped trajectory with 30,000 configurations for 5000 molecules and employed a total of 300 basis functions to represent the pair and LD potentials. While the calculation required 19.3 h on a single processor, it required only 24 min on 96 processors. We find that the time, *t*_n_, required for performing this calculation on n processors is very accurately described by the simple function *c*_1_ + *c*_2_/n with *c*_1_ = 0.199 h and *c*_2_ = 19.1 h. Thus, it appears that 99% of the total computational effort is spent computing *G*_*DD*′_ and *b*_*D*_. This fraction of the effort scales very well. Conversely, approximately 1% is fixed by setting up the calculation, solving the normal equations, and reporting the results.

**Figure 2:**
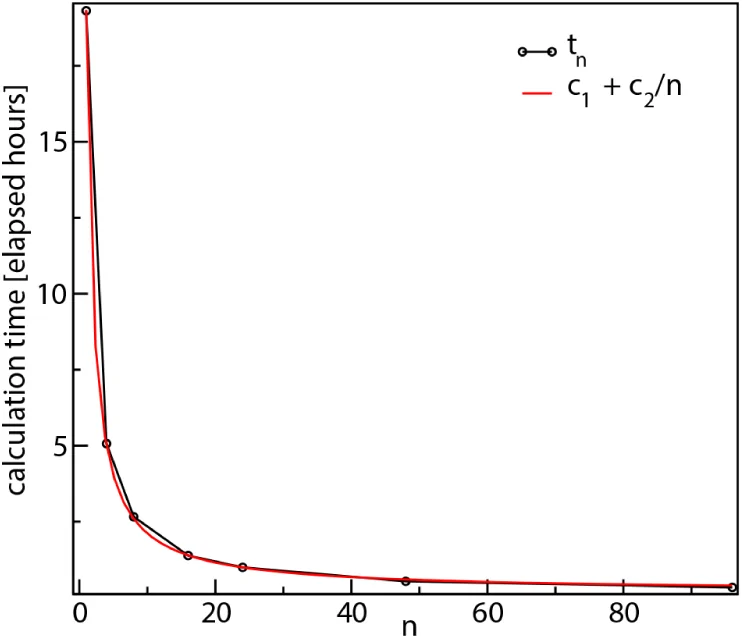
Scaling of the force-matching calculation for parameterizing the one-site LD-BULK water model in Section 4.2. The black curve reports the time elapsed, *t*_n_, when performing the force-matching calculation with n processors. The red curve plots *c*_1_ + *c*_2_/n with *c*_1_ = 0.199 h and *c*_2_ = 19.1 h.

BOCS employs its own file and data structures for treating topology and trajectory information. Because it was initially developed from GROMACS software,^82–84^ BOCS originally enjoyed native compatibility with GROMACS topology and trajectory files, e.g., binary .tpr and .trr files. Subsequently, we developed stand-alone file formats and data structures that no longer rely upon GROMACS installations. BOCS now treats CG topology information with a compact, human-readable .btp file format that is quite similar to a dumped .tpr file. While it can directly interface with conventional trajectory files from GROMACS and LAMMPS, BOCS employs a text, human-readable .btj file to store the LD information in the extended mapped trajectory. Additionally, BOCS can read LAMMPS trajectory files that were supplemented with LD information.

We developed a translator tool for porting topology and trajectory information into the BOCS format. The translator tool can convert dumped .tpr files from GROMACS into .btp format. The translator tool can also convert GROMACS and LAMMPS trajectory files into the .btj format, as well as interconvert between these trajectory formats. This tool provides BOCS with considerable flexibility while still enjoying the benefits of the GROMACS data structures. We anticipate regularly updating the translator tool to maintain compatibility with future versions of the packages.

Finally, BOCS also provides a variety of tools for analysis and for facilitating simulations. For instance, the LDPDF tool computes and outputs the sampled distribution for each type of local density and its gradient. Similarly, the cgff tool employs the g-YBG framework to analyze the cross-correlations among different degrees of freedom and to quantify their contribution to the calculated potential. We have also developed new Python scripts, LD_table_prep_from_spline.py and SG_table_prep_from_spline.py, that format the calculated LD and SG potentials for simulations in LAMMPS.

#### 3.1.3 Weighting Functions

The local density, ρ_*I*_, around each particle, *I*, is defined by a normalized weighting function, *w̅*(*r*), according to eq 6. We have implemented 5 different weighting functions into BOCS and LAMMPS. Each function is determined by a non-increasing function, *w*(*r*), that decreases from 1 to 0 over the interval 0 ≤ *r* ≤ *r*_c_ and vanishes for *r* ≥ *r*_c_. Each function is then normalized such that $\int_{0}^{r_{c}}4\pi r^{2}dr\mathrm{w̅}\left(r\right)=1$ . The SI provides explicit expressions for each function.

Figure 3a plots the 5 normalized weighting functions, *w̅*(*r*). The dashed red curve plots the quadratic weighting function that is often employed in dissipative particle dynamics (DPD) models.^51,85,86^ The dashed blue curve plots the Lucy weighting function^87,88^ that has been used in several MS-CG models with LD and SG potentials.^54,56,57,68,72^ These weighting functions both decrease over the entire interval 0 ≤ *r* ≤ *r*_c_. The remaining weighting functions are constant over an inner interval 0 ≤ *r* ≤ *r*_0_ and then decrease from *r*_0_ ≤ *r* ≤ *r*_c_. For concreteness, we have set *r*_0_ = *r*_c_/2 in Figure 3. The dotted green curve plots the weighting function employed by Sanyal and Shell.^62,64^ The dotted cyan curve presents the “sphere” weighting function, which quantifies the fraction of overlap between two spheres that are separated by a distance *r*. Finally, the thin pink curve presents a weighting function that decays more smoothly.

**Figure 3:**
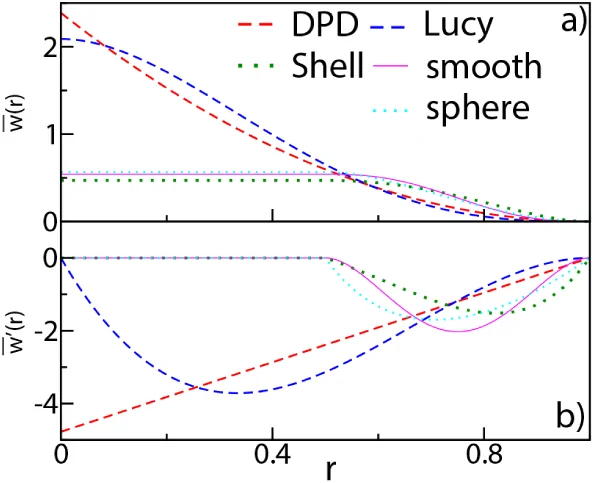
Normalized weighting functions implemented in BOCS and LAMMPS. We have plotted *w̅*(*r*) and *w̅*′(*r*) in dimensionless units by rescaling distances with respect to *r*_c_, i.e., *r* → *r*/*r*_c_. We have set *r*_0_ = *r*_c_/2 for the Shell, smooth, and sphere weighting functions.

Figure 3b plots the corresponding derivatives, *w̅*′(*r*), that directly contribute to the forces in eq 10. This derivative vanishes at *r*_c_ for all weighting functions in order to ensure that the LD force is continuous. However, the higher-order derivatives distinguish the various weighting functions. The second derivative of the Lucy weighting function vanishes at *r*_c_ and is continuous for all *r*. In contrast, the second derivative of the DPD weighting function has a constant finite value over the interval 0 ≤ *r* < *r*_c_, but is discontinuous at *r*_c_. Similarly, the Shell and sphere weighting functions have discontinuous second derivatives at both *r*_0_ and *r*_c_. The smooth weighting function has continuous second derivatives for all *r* that vanish for *r* ≤ *r*_0_ and *r* ≥ *r*_c_.

### 3.2 Testing the New BOCS

We report three tests that validate our implementation of LD and SG potentials in LAMMPS and BOCS. In each test, we employed PKG-BOCS to simulate a known interaction potential, *U*_R_(**R**), in LAMMPS. We then employed BOCS to perform the variational force-matching calculation with the simulated configurations and net forces. All force-matching calculations employed BOCS version 5.0, which is available on the Noid group GitHub page.^80^ All LAMMPS calculations employed the version that is hosted on the Noid group GitHub page at noid-group/lammps-BOCS2026.^79^ Specifically, we created a branch, “BOCS-2026LD”, within this repository to distribute the src code and documentation for the reported calculations. We have obtained equivalent results for all three tests with the final version of PKG-BOCS that is included in the 4 July 2026 LAMMPS release, which is distributed at https://github.com/lammps/lammps.

Each LAMMPS simulation employed a Verlet neighbor list and three-dimensional periodic boundary conditions. We employed a Nosé–Hoover chain thermostat^89–91^ when simulating the first and third test systems. We employed a Langevin thermostat^92,93^ when simulating the second test system with only two particles. We set the inner cutoff of the Shell, sphere, and smooth weighting functions to *r*_0_ = 0 for simplicity. Each force-matching calculation solved the corresponding normal equations via singular value decomposition with the hyperparameters listed in the SI. As discussed in Section 2.3, if the basis functions describing pair and LD potentials are sufficiently flexible, then there exists a family of potentials that generate identical forces. In particular, there exists a family of pair and LD potentials that all minimize χ^2^. We report the member of this family that most nearly matches the simulated potentials. We identify this member based upon an automated procedure previously described in refs 58 and 70. The SI describes these benchmark tests in much greater detail.

### 3.3 Applying the New BOCS

We illustrate this new software by developing and assessing 1-site CG water models based upon the AA SPC/E model.^94^ We refer readers to our recent study^70^ for a much more detailed analysis of CG water models based upon the 4-site TIP4P model.^95^ As described above in Section 3.2, we performed all force-matching calculations with BOCS version 5.0, which is available on the Noid group GitHub page.^80^ Similarly, we performed all LAMMPS simulations with the “BOCS-2026LD” version that is available on the Noid group GitHub page.^79^

#### 3.3.1 SPC/E Model

We performed two simulations of *N* = 5000 water molecules with GROMACS/2019.6, while propagating dynamics with the md leapfrog integrator, a 2 fs time step, and periodic boundary conditions in all 3 directions.^82–84^ We described intermolecular interactions with the SPC/E water model,^94^ while employing the LINCS^96^ algorithm to rigidly constrain the geometry of each molecule. We treated long-range electrostatic interactions with the particle mesh Ewald (PME) method,^97^ while truncating dispersive interactions and short-range PME contributions at 1.55 nm. We did not treat dispersion corrections in order to ensure consistency between bulk and slab simulations. We sampled energy fluctuations with the Bussi thermostat^98^ while employing a 0.5 ps relaxation time. We sampled volume fluctuations with the Parrinello–Rahman^99^ barostat while employing a relaxation time of 5 ps and a compressibility of 4.5 × 10^–5^ bar^–1^.

We first simulated bulk water in the constant NPT ensemble under ambient conditions: *T*_0_ = 300 K and *P*_0_ = 1 bar. We constructed initial conditions for this NPT simulation by employing gmx insert-molecules to insert *N* = 5000 water molecules into a cubic box with sides of length *L* = 5.32502 nm and conducting a brief energy minimization. We simulated this configuration for 34 ns and treated the first 4 ns as an equilibration. We extended the z-dimension of the last configuration from this constant NPT simulation to *z* = 16.0 nm. We then simulated the resulting configuration in the constant NVT ensemble at *T*_0_ = 300 K for 34 ns and treated the first 4 ns as an equilibration. We sampled each production simulation every 500 timesteps, corresponding to a rate of 1 frame/ps.

After performing the bulk and slab simulations, we then simulated a film of SPC/E water in a fixed box with dimensions *L*_*x*_ = *L*_*y*_ = 5.3189 nm and *L*_*z*_ = 25 nm. We confined the molecules in the z-dimension by employing an external field, *V*(*z*), that acted on a virtual site located at the mass center of each molecule. This external field included two components, *V*(*z*) = *V*_lt_(*z*) + *V*_rt_(*L*_*z*_ – *z*), which described an attractive wall on the left and a repulsive wall on the right, where *z* is the z-coordinate of the virtual site. The potential for the repulsive right wall, *V*_rt_(*z*), was adopted from ref 57 without modification. The potential for the attractive left wall, *V*_lt_(*z*), is a scaled version of the original potential, *ν*_lt_(*z*), in ref 57, *V*_lt_(*z*) = *ν*_lt_(2*z*)/2. We provide the explicit form of this external field in the accompanying data repository.^100^ We implemented the external field via a table file with the GROMACS group cutoff scheme, while employing two-dimensional periodic boundary conditions in the x and y directions. These simulations employed the same time step and neighbor list parameters as the bulk production simulation, while also neglecting dispersion corrections. However, we modified the PME calculation to account for the finite z-dimension by employing the ewald-geometry = 3dc directive.

We constructed an initial configuration for this simulation by inserting 5000 molecules into a cubic box with sides of length *L* = 5.3189 nm. We extended the z-dimension of the simulation box to *L*_*z*_ = 25 nm and translated each molecule by 0.5 nm from the left boundary. We simulated this initial configuration for 5 ns while employing the Berendsen thermostat^101^ to approach equilibrium at *T* = 300 K. We then simulated the final configuration for 20 ns while employing the Bussi thermostat with a 0.5 ps relaxation time to sample the constant NVT ensemble at *T* = 300 K. We discarded the first 2 ns as additional equilibration and analyzed the remaining 18 ns of the simulation.

#### 3.3.2 CG Models

We parameterized three different 1-site CG water models from these SPC/E simulations via variational force-matching, while employing the default settings to solve eq 22.

The first two CG models both employed the same pair-additive form for the approximate CG potential, *U*(**R**) = ∑_(*I*,*J*)_ *U*_2_(*R*_*IJ*_). We represented the pair force function, *F*_2_(*r*), in each case with 4th order (cubic) splines, while employing a knot spacing of δ*r* = 0.02 nm over the interval 0.0 nm ≤ *r* ≤ 1.4 nm. We parameterized the PAIR-BULK and PAIR-SLAB models based upon the bulk and slab SPC/E simulations, respectively.

The third CG model included both pair and LD terms in the approximate potential, $U\left(R\right)=\underset{\left(I,J\right)}{\sum }U_{2}\left(R_{IJ}\right)+\overset{N}{\underset{I=1}{\sum }}U_{\rho }\left(\rho_{I}\right)$ . We parameterized this LD-BULK model from the bulk SPC/E simulation. We represented the pair force function, *F*_2_(*r*), with 4th-order (cubic) splines, while employing a knot spacing of δ*r* = 0.01 nm over the interval 0.0 nm ≤ *r* ≤ 1.4 nm. We defined the local density, ρ_*I*_, around each site, *I*, with a normalized Lucy function, while including the self-term in eq 6. We employed an automated search^70^ to identify the LD length-scale, *r*_c_ = 0.392 nm, that best reproduced the bulk density of the SPC/E model. We represented the LD force function, *F*_ρ_(ρ_L_), with 4th-order (cubic) splines, while employing a knot-spacing δρ_L_ = 0.1 nm^–3^ over the range of local densities sampled by the bulk SPC/E simulation. We extrapolated *F*_ρ_(ρ_L_) outside of this range according to the automated procedure previously described in ref 70. Briefly, we identified the domain of the calculated LD force function for which the AA simulation provided adequate statistics. Near each boundary of this adequately sampled domain, we fit the calculated force to a quadratic function. We ensured that the quadratic fit for low local densities, ρ_L_ → 0, had positive curvature in order to avoid artificially destabilizing the liquid phase. We then employed these quadratic fits to extrapolate the LD force function for high and low local densities that were rarely sampled by the AA simulation.

We simulated these CG models with LAMMPS, while propagating dynamics with a 2 fs time step. We performed bulk and slab simulations of each CG model with three-dimensional periodic boundary conditions. We also simulated each CG model in the presence of the external field described above, while employing 2-dimensional periodic boundary conditions in the *x*- and *y*-directions. This external field acted directly upon the z-coordinate of each CG site. Constant NVT simulations employed a Nosé–Hoover chain thermostat^89–91^ while employing a 200 fs relaxation time and the default chain length *n* = 3. Constant NPT simulations employed the equations of motion developed by Shinoda et al.^102–104^ All interactions were truncated at a cutoff *r*_cut_ = 1.4 nm. We employed a neighbor list with a cutoff of *r*_list_ = 3 nm, which was checked at every time step. While this unnecessarily long neighbor list reduced the efficiency of our CG simulations, it did not impact their accuracy. We obtained the initial configurations for these simulations by mapping the last configuration of the corresponding SPC/E simulation to the CG resolution.

## 4 Results and Discussion

### 4.1 Benchmark Tests

In this subsection, we present three benchmark tests that illustrate and validate our implementation of LD and SG potentials in LAMMPS and BOCS. In each case, we first simulated a known potential, *U*_sim_, in LAMMPS with the PKG-BOCS package. We then employed BOCS to calculate a potential, *U*_FM_, for the same resolution via variational force-matching with the simulated configurations and forces. Because we are not coarse-graining the underlying model, the known simulated potential minimizes the force-matching functional, i.e., χ^2^[*U*_sim_] = 0. Consequently, the difference, *U*_sim_(**R**) – *U*_FM_(**R**), between the simulated and calculated potentials directly assesses the software that we implemented in LAMMPS and BOCS. In addition to demonstrating the accuracy of our implementation, these studies provide insight into the forces generated by LD and SG potentials. In particular, we highlight the impact of the LD weighting function, *w̅*, upon these forces.

#### 4.1.1 Test 1:400 Particles with Linear LD Potential

We first consider a system of *N* = 400 spherical particles that interact only via LD potentials:

23 $$U\left(R\right)=\overset{N}{\underset{I=1}{\sum }}U_{\rho }\left(\rho_{I}\right)$$

We adopt the simplest possible LD potential,

24 $$U_{\rho }\left(\rho_{L}\right)=-k\rho_{L}$$

with *k* = 0.44504, which is plotted in the inset of Figure 4a. Here, we employ reduced units that correspond to scaling energies by the thermal energy, *k*_*B*_ *T*, and scaling distances with respect to the LD length-scale, *r*_c_. The inset of Figure 4b presents the corresponding LD force function, *F*_ρ_(ρ_L_) = −d*U*_ρ_(ρ_L_)/dρ_L_ = +*k*. The LD force function is independent of ρ_L_ and always drives particles toward regions of higher local density. Because eq 23 does not account for excluded volume, this interaction potential cannot realistically model molecular systems. Nevertheless, the LD potential defined by eq 24 may be useful for reducing the internal pressure of a more realistic potential that already treated excluded volume. Moreover, the simplicity of the present model provides transparent insight into the forces generated by LD potentials.

**Figure 4:**
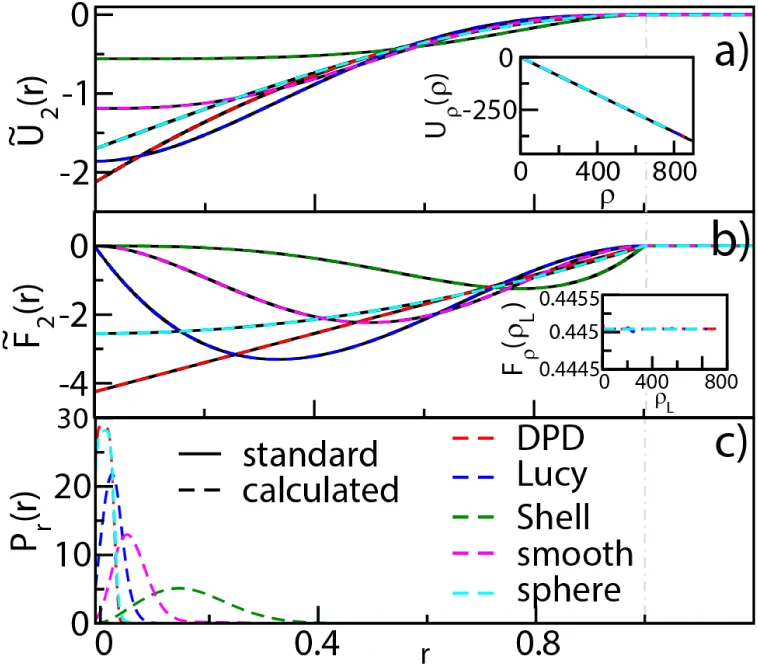
Analysis of *N* = 400 molecules interacting via only LD potentials. The insets of panels a and b report the simulated LD potential, *U*_ρ_, and force function, *F*_ρ_. The remainder of panels a and b report the corresponding effective pair potential, *Ũ*_2_, and pair force, *F̃*_2_. Panel c reports the simulated pair distributions. The red, blue, green, pink, and cyan curves correspond to the DPD, Lucy, Shell, smooth, and sphere weighting functions, respectively. In panels a and b, the solid black curves present the standard functions that are defined by the simulated potential, *U*, while the dashed colored curves present the potentials and force functions determined via variational force-matching. We have scaled energies with respect to *k_B_T* and distances with respect to *r*_c_.

The definition of the local density in eq 6 implies that, if *U*_ρ_(ρ_L_) is an *m* ^th^ degree polynomial in ρ_L_, then *U*_ρ_ can be represented as a sum of *m* + 1-body terms.^86^ Consequently, the total potential, eq 23, can be re-expressed in terms of central pair potentials:

25 $$U\left(R\right)=\underset{\left(I,J\right)}{\sum }{Ũ}_{2}\left(R_{IJ}\right)$$

where

26 $${Ũ}_{2}\left(r\right)=-2k\mathrm{w̅}\left(r\right)$$

Figure 4a and b present the corresponding pair potentials, *Ũ*_2_(*r*), and pair forces, *F̃*_2_(*r*) = −d*Ũ*_2_(*r*)/d*r*, for the 5 weighting functions that we have implemented in BOCS and LAMMPS. (We have set the inner radius of the Shell, smooth, and sphere weighting functions to *r*_0_ = 0 for simplicity.) These curves emphasize that the interaction potential depends not only upon the LD potential, *U*_ρ_, but also on the weighting function used to define the local density. Given the fixed LD potential function, the DPD and Lucy weighting functions result in the most attractive pair potentials, while the Shell weighting function results in the least attractive potential. The pair forces for the Shell, smooth, and Lucy weighting functions are most attractive at *r* ≈ 0.80, 0.55, and 0.37, respectively, and all vanish at *r* = 0. Conversely, the pair forces for the DPD and sphere weighting functions become increasingly attractive as *r* decreases and exert the largest forces at *r* = 0.

We employed LAMMPS to simulate a system of *N* = 400 particles interacting with the LD potential *U*_ρ_(ρ_L_) = −*k*ρ_L_, while employing each of the five weighting functions described in Section 3.1.3. Figure 4c presents the sampled distribution, *P*_r_(*r*), of pair distances for each of the five simulations. The simulated statistics follow the expected trends. In particular, the DPD and sphere weighting functions result in the most compact aggregates, while the Shell weighting function results in more extended aggregates.

We employed BOCS to determine the simulated force functions via variational force-matching. We performed this force-matching calculation with two different basis sets. We first employed a basis set corresponding to local density forces. The inset of Figure 4b demonstrates that BOCS quantitatively recovered the same simulated LD force function, *F*_ρ_(ρ_L_) = +*k*, from each of the 5 simulations. We then employed a basis set corresponding to simple pair potentials. The main panel of Figure 4b demonstrates that, in this case, BOCS quantitatively recovered the corresponding pair force for each of the 5 weighting functions.

#### 4.1.2 Test 2: Two Particles with LD or SG Potentials

##### 4.1.2.1 LD Potential

We next consider a system of *N* = 2 spherical particles that interact only via LD potentials according to eq 23. In this second test, we consider the slightly more complex quadratic LD potential,

27 $$U_{\rho }\left(\rho_{L}\right)=\frac{1}{2}k{\left(\rho_{L}-\rho_{*}\right)}^{2}$$

which is plotted in Figure 5a. The corresponding LD force function, *F*_ρ_(ρ_L_) = −d*U*_ρ_(ρ_L_)/dρ_L_ = −*k*(ρ_L_ – ρ_*_) generates attractive forces when ρ_L_ < ρ_*_ and repulsive forces when ρ_L_ > ρ_*_. We have again scaled energies with respect to *k*_*B*_ *T* and distances with respect to *r*_c_.

**Figure 5:**
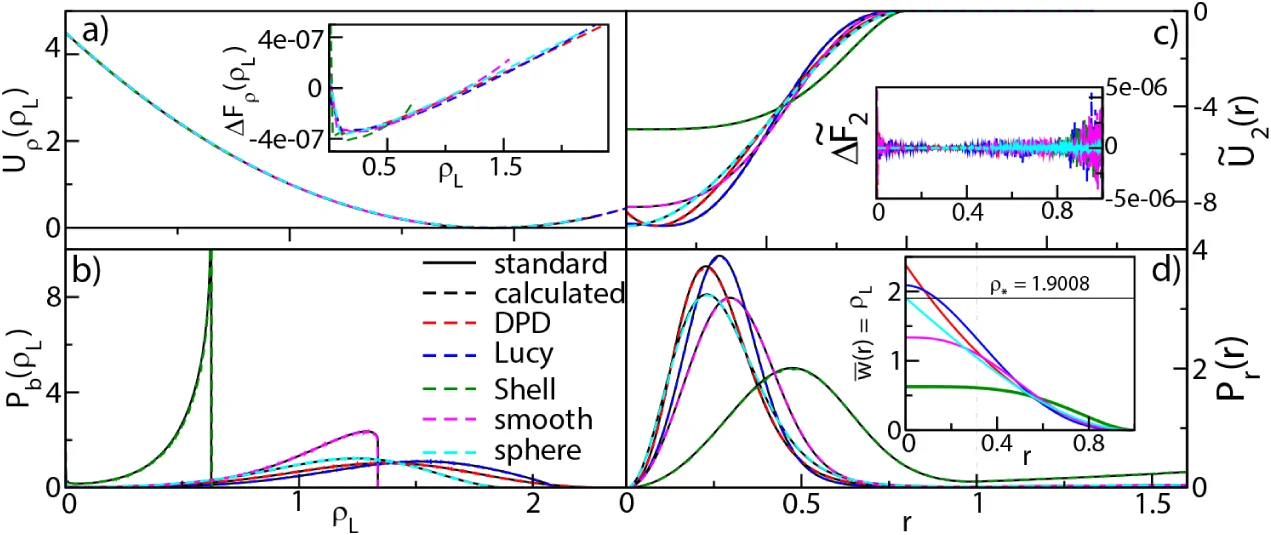
Analysis of *N* = 2 particle simulations employing the LD potential defined by eq 27. Panel a presents *U*_ρ_(ρ_L_), while panel b presents *P*_b_(ρ_L_). Panel c presents *Ũ*_2_(*R_IJ_*), while panel d presents *P*_r_(*r*). In panels a and c, the solid black curves present the standard LD and pair potentials that are defined by the simulated potential, while the dashed curves present the corresponding potentials calculated via variational force-matching. The insets in these panels report the errors in the force-matching calculations. In panels b and d, the solid black curves present the analytically determined, canonical probability distributions, while the dashed colored curves present the simulated distributions. The inset in panel d presents the 5 local density weighting functions, *w̅*, while the horizontal line indicates the equilibrium local density, ρ_*_. The color scheme and scaling are retained from Figure 4.

In the present case of *N* = 2 particles, both particles have the same local density, ρ_1_ = ρ_2_ = ρ_L_, which is determined by the weighting function, ρ_L_ = *w̅*(*R*_12_). The inset of Figure 5d presents this relationship for the 5 weighting functions implemented in BOCS and LAMMPS. (We have again set *r*_0_ = 0 for the Shell, smooth, and sphere weighting functions.) When the particles are separated by *R*_12_ > 1, the local density vanishes, ρ_L_ = *w̅*(*R*_12_) = 0. Conversely, over the distance range 0 ≤ *R*_12_ ≤ 1, ρ_L_ increases as the particles approach and attains a maximum, ρ_max_, at *R*_12_ = 0. Because the weighting functions have different shapes and, thus, different normalizations, the maximum local density differs among the weighting functions. In the case of the DPD and Lucy weighting functions, the local density can exceed ρ_*_ when particles are sufficiently close, i.e., ρ_L_ > ρ_*_ for *R*_12_ < r_*_. In contrast, the local density is always less than ρ_*_ for the Shell, smooth, and sphere weighting functions.

In general, a quadratic LD potential will generate 3-body forces. However, for the present case of *N* = 2 particles, the total potential, *U*, can be expressed in terms of a single effective pair potential, *Ũ*_2_, that explicitly reflects the weighting function:

28 $${Ũ}_{2}\left(R_{IJ}\right)=k{[\mathrm{w̅}(R_{IJ})-\rho_{*}]}^{2}$$

Figure 5c presents these pair potentials for the 5 weighting functions. These pair potentials appear quite similar to the corresponding potentials in Figure 4a. The greatest discrepancies arise for the DPD and Lucy weighting functions. In these cases, the pair potential generates repulsive forces at sufficiently small distances, *R*_12_ < r_*_, for which ρ_L_ > ρ_*_. In contrast, the pair potentials are never repulsive for the Shell, smooth, and sphere weighting functions.

It is straightforward to analytically characterize the equilibrium Boltzmann distribution for *N* = 2 particles. Figure 5d plots the canonical probability density, *P*_r_(*r*) ∝ *r* ^2^ exp­[−β*Ũ*_2_(*r*)], for the two particles to be separated by a distance *r*. The probability densities for the DPD and Lucy weighting functions are both sharply peaked near the distance r_*_ corresponding to ρ_*_. Conversely, the Shell weighting function results in a much broader distribution that is peaked at considerably larger distances and, moreover, gives considerable weight to dissociated configurations with *r* > 1. The relationship ρ_L_ = *w̅*(*r*) then determines the corresponding local density distribution, *P*_ρ_(ρ_L_) = *P*_f_δ­(ρ_L_) + *P*_b_(ρ_L_), where *P*_f_ is the total probability for dissociated configurations, while *P*_b_(ρ_L_) is the equilibrium probability density for bound configurations. Figure 5b presents *P*_b_(ρ_L_), which necessarily vanishes for ρ_L_ > *w̅*(0). This distribution reflects not only the Boltzmann weight, exp­[−β*U*_ρ_(ρ_L_)], associated with the LD potential, but also the Jacobians associated with transforming from Cartesian coordinates to the pair distance, *R*_12_, and then to the local density, ρ_L_. Consequently, because it varies least rapidly with *r*, the Shell weighting function results in the broadest *r* distribution and the sharpest ρ_L_ distribution. Clearly, the equilibrium distribution depends quite sensitively upon the specific form of the local density weighting function, *w̅*(*r*).

We performed 5 independent Langevin dynamics simulations that sampled the canonical ensemble for the LD potential in eq 27 with each of the 5 weighting functions. The dashed curves in Figure 5c and d report the sampled distance and LD distributions, respectively. The quantitative agreement between the analytically determined canonical distributions and the simulated distributions demonstrates the accuracy of our implementation of LD potentials in LAMMPS.

We then employed BOCS to determine the simulated force functions from the forces sampled by each of the 5 trajectories. As in the preceding example, we performed two independent variational FM calculations for each trajectory. The first FM calculation represented the forces with an LD force function, while the second represented the forces with a central pair force function. The insets of Figure 5a and c report the errors in the calculated LD and pair force functions. BOCS again determines these force functions very accurately. In particular, the errors in the calculated pair force are very small and fluctuate around 0. The errors in the calculated LD force are also very small and reflect the boundary conditions of the spline functions employed in the variational FM calculations. Specifically, while the calculated LD force function necessarily vanishes for the largest and smallest local densities, Figure 5a indicates that the simulated LD force function does not.

##### 4.1.2.2 SG Potential

We now consider a system of *N* = 2 spherical particles that interact only via SG potentials:

29 $$U\left(R\right)\equiv \overset{N}{\underset{I=1}{\sum }}U_{\nabla }\left(\rho_{I}\right){|\nabla_{I}\rho_{I}|}^{2}=2U_{\nabla }\left(\rho_{L}\right){|\nabla \rho_{L}|}^{2}$$

The second expression simplifies *U* for the case of *N* = 2: ρ_L_ ≡ ρ_1_ = ρ_2_ and **∇**ρ_L_ ≡ **∇**_1_ρ_1_ = –**∇**_2_ρ_2_. Classical density functional theory and phenomenological mean field theories often describe the free energy cost of density gradients with similar functional forms where the coefficient of the square gradient is a positive constant, i.e., *U*_**∇**_(ρ_L_) = κ > 0.^71,78^ BOCS allows a more general treatment of the square gradient coefficient, *U*_**∇**_(ρ_L_), that can be tuned to act specifically at interfaces.^72^

We consider a linear SG coefficient function for this illustration,

30 $$U_{\nabla }\left(\rho_{L}\right)\equiv k\left(\rho_{L}-\rho_{*}\right)$$

which is plotted in Figure 6a. Note that *U*_**∇**_ is not a potential and has dimensions of $k_{B}T/r_{c}^{8}$ . (We have again scaled energies and distances with respect to *k*_*B*_ *T* and *r*_c_, respectively.) Because *U*_**∇**_(ρ_L_) < 0 over the relevant density range, *U* stabilizes density gradients.

**Figure 6:**
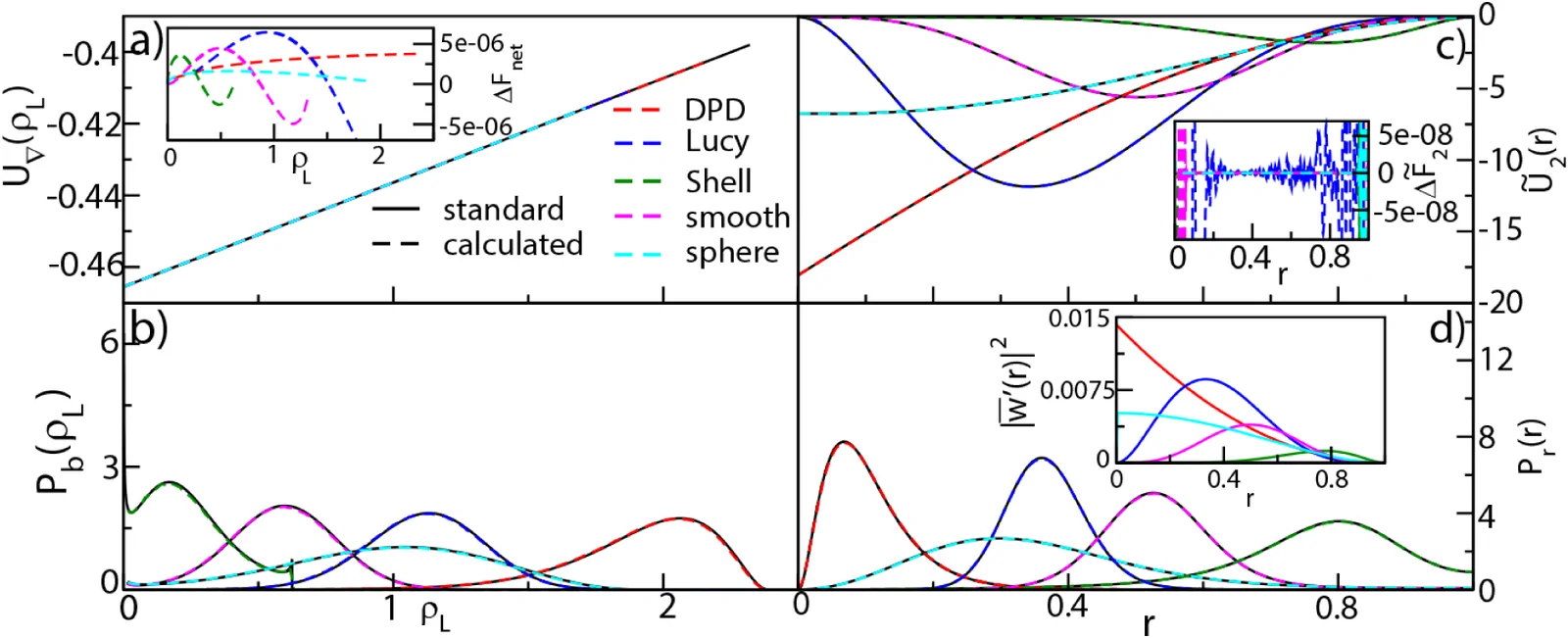
Analysis of *N* = 2 particle simulations employing the SG potential defined by eq 29. Panel a presents *U* **_∇_** (ρ_L_), while panel b presents *P*_b_(ρ_L_). Panel c presents *Ũ*_2_(*r*), while panel d presents *P*_r_(*r*). In panels a and c, the solid black curves present the standard SG coefficients and pair potentials that are defined by the simulated potentials, while the dashed curves present the corresponding functions calculated via variational force-matching. The insets in these panels present the errors in the force-matching calculations. In particular, the inset of panel a presents the error in the net force on each particle resulting from the force-matching calculation with the SG basis set, while the inset of panel b presents the error in the calculated pair force function. In panels b and d, the solid black curves present the analytically determined, canonical probability distributions, while the dashed colored curves present the simulated distributions. The inset in panel d presents |*w̅*′(*r*)|^2^. The color scheme and scaling are retained from Figure 4.

For the present case of *N* = 2 particles, |**∇**ρ_L_|^2^ = |*w̅*′(*R*_12_)|^2^. While the inset of Figure 5d plots ρ_L_ = *w̅*(*R*_12_) for the 5 weighting functions implemented in BOCS, the inset of Figure 6d plots the corresponding square gradients, |*w̅*′(*R*_12_)|^2^, as a function of the pair distance, *R*_12_ = *r*. In the case of the DPD and sphere weighting functions, |*w̅*′(*r*)|^2^ monotonically increases as *r* decreases and approaches its maximum as *r* → 0. However, |*w̅*′(*r*)|^2^ is significantly smaller and varies less rapidly with distance for the sphere weighting function. Conversely, |*w̅*′(*r*)|^2^ attains a maximum at a finite distance *r*_**∇**_ ≈ 0.335, 0.5, and 0.775 for the Lucy, smooth, and Shell weighting functions, respectively.

For the case of *N* = 2 particles, the SG potential can be expressed in terms of a central pair potential, *U*(**R**) = *Ũ*_2_(*R*_12_), with

31 $${Ũ}_{2}\left(r\right)=2U_{\nabla }\left(\mathrm{w̅}\left(r\right)\right){|{\mathrm{w̅}}^{\prime }\left(r\right)|}^{2}$$

which is plotted in Figure 6c. By stabilizing local density gradients, the SG potential generates an attraction between the pair. The corresponding force function is

32 $${\mathrm{F̃}}_{2}\left(r\right)=-d{Ũ}_{2}\left(r\right)/dr=2{\mathrm{w̅}}^{\prime }\left(r\right)\left[F_{\nabla }\left(\mathrm{w̅}\left(r\right)\right){|{\mathrm{w̅}}^{\prime }\left(r\right)|}^{2}-2U_{\nabla }\left(\mathrm{w̅}\left(r\right)\right){\mathrm{w̅}}^{\prime \prime }\left(r\right)\right]$$

Note that eq 32 includes terms that depend upon both *U*_**∇**_ and *F*_**∇**_ = −d*U*_**∇**_/dρ_L_ in eq 32. Appendix 2 demonstrates that, for *N* = 2, one can shift *U*_**∇**_(ρ_L_) → *Ũ*_**∇**_(ρ_L_) = *U*_**∇**_(ρ_L_) + *c*|**∇**ρ_L_|^–2^ (for an arbitrary constant *c*) without impacting the simulated forces.

Figure 6c demonstrates that the SG potential depends very strongly upon the shape of the weighting function. In particular, the potential for the DPD weighting function is always attractive and becomes increasingly attractive as particles approach. The potential for the sphere weighting function is also always attractive but much weaker. In contrast, the potentials for the Lucy, smooth, and Shell weighting functions switch from attractive to repulsive near the distance, *r*_**∇**_, that maximizes |**∇**ρ_L_|^2^.

The solid curves in Figure 6b and d present, for each weighting function, the analytically determined canonical probabilities for local densities, *P*_b_(ρ_L_), and for pair distances, *P*_r_(*r*), respectively. These distributions are consistent with the preceding discussion. The distance and LD distributions for the DPD weighting function are peaked near *r*_**∇**_ = 0 and the corresponding local density, ρ_max_, respectively. The distance distributions for the Lucy, smooth, and Shell weighting functions are all peaked near the corresponding distances *r*_**∇**_ > 0, while the LD distributions are peaked near the corresponding local density, ρ_L**∇**_ = *w̅*(*r*_**∇**_). Because |**∇**ρ_L_|^2^ varies less rapidly with distance for the sphere weighting function, the corresponding distributions are much broader.

The dashed curves in Figure 6b and d present the distributions sampled by 5 independent Langevin dynamics simulations with the potential, *U*(**R**), defined by eqs 29 and 30 for the 5 different weighting functions. The quantitative agreement between the canonical and simulated distributions again illustrates the accuracy of the LAMMPS implementation of the SG potentials.

We employed BOCS to determine the simulated potentials from the forces sampled by each of the 5 trajectories. The dashed curves in Figure 6a and c present the SG coefficient functions and pair potentials obtained from independent force-matching calculations with the corresponding basis functions. We again observe that BOCS determines the simulated potentials with quantitative accuracy. The insets of these panels present a more quantitative assessment of these errors. The inset of Figure 6a reports the error in the net forces generated by the calculated SG coefficient function. This error is very small, fluctuates about 0, and reflects the relationship between *U*_**∇**_ and *F*_**∇**_. The inset of Figure 6b demonstrates that the error in the calculated pair force is similarly small, fluctuates about 0, and reflects limited sampling and the boundary conditions placed upon the spline calculations.

#### 4.1.3 Test 3:125 Molecules with LD and SG Potentials

Our third test considers a much more complex system of 125 molecules. Each molecule consists of 9 particles of 4 distinct types (τ = A, B, C, or D) that are connected in a branched topology, as illustrated in the inset of Figure 7. The B and C sites are located at the two branch points, while the A and D sites are located at the surface of the molecule.

**Figure 7:**
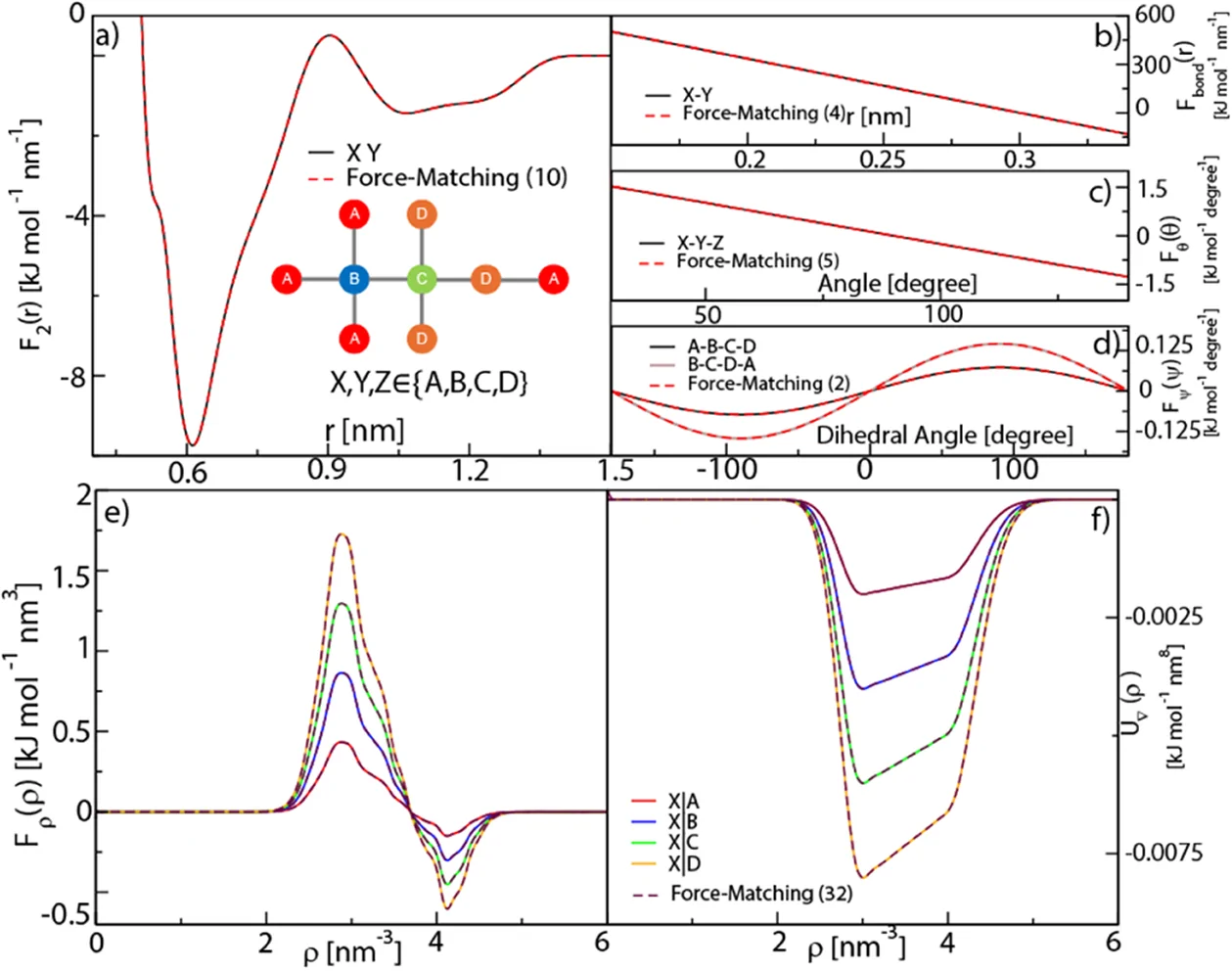
Forces governing the third test. Panel a presents the pair force function describing all nonbonded pair interactions, while the inset illustrates the connectivity of each molecule. Panels b, c, and d present the bond, angle, and dihedral force functions, respectively. Panels e and f present the LD force functions and SG coefficient functions, respectively. The solid black curves present the standard (i.e., ground truth) functions defined by the simulated potential, while the dashed colored curves present the corresponding functions calculated via variational force matching.

The bonded potential included 4 distinct bond potentials to govern the A–B, B–C, C–D, and D–A bonds; 5 distinct angle potentials to govern the A–B–A, A–B–C, B–C–D, C–D–A, and D-C–D angles; and 2 distinct dihedral potentials to govern the A–B-C–D and B–C–D-A dihedrals. Given the four distinct site types, the nonbonded potential was defined by 10 central pair potentials, 16 LD potentials, and 16 SG coefficient functions. In addition to governing intermolecular interactions, the nonbonded potential also governed intramolecular 1–5 interactions between A sites on opposite sides of the molecule; that is, we excluded intramolecular 1–2, 1–3, and 1–4 interactions from the nonbonded potential.

We defined the 53 distinct potentials with 13 different functions in order to simplify the presentation. Figure 7b and c presents the linear functions that defined the bond and angle forces, respectively. Figure 7a presents the function that was used to define all nonbonded pair forces. This pair force includes a repulsive hard core region for *r* < 0.507 nm, as well as attractive regions over the intervals 0.507 nm < *r* < 0.863 and 0.950 nm < *r* < 1.50 nm. Moreover, while this model treated 16 distinct types of local density, we adopted the same Lucy weighting function with *r*_c_ = 0.65 nm to define the local density, ρ_τ|*I*_, of τ type sites around each site, *I*. (In practical calculations, it may be beneficial to employ a different length-scale for each type of site or local density because the accuracy of CG models can be quite sensitive to *r*_c_.^56,58,59^ ) Similarly, we assumed that the LD potentials, *U*_ρτ|_ _τ_*I*__(ρ_τ|*I*_), and SG coefficient functions, *U*_**∇**τ|τ_*I*__(ρ_τ|*I*_), depended only upon the type, τ_*I*_, of the central site, *I*, and not upon the type, τ, of the surrounding sites. Figure 7e and f presents the LD force functions and SG coefficient functions, respectively. The LD potentials have a single minimum at ρ_L_ = 3.7 nm^–3^ and only generate forces in the interval 1.63 nm^–3^ < ρ_L_ < 5.35 nm^–3^. The SG coefficient functions stabilize density gradients when 1.91 nm^–3^ < ρ_L_ < 5.44 nm^–3^ and vanish outside of this interval. Furthermore, the LD potential and SG coefficient functions for different site types are related by scalar multiples. The SI describes the interaction potential in greater detail.

We employed LAMMPS to simulate the system at a constant temperature, *T* = 350 K, in a fixed cubic box with sides of length, *L* = 4 nm, and periodic boundary conditions in all 3 directions. Starting from the random homogeneous configuration shown in Figure 8a, the system quickly evolved to form the porous two-dimensional sheet shown in Figure 8b that is roughly parallel to the *y*–*z* plane. SI demonstrates that the simulated morphology reflects the periodic boundary conditions, as the 125 molecules form an approximately spherical drop when simulated in a sufficiently large volume.

**Figure 8:**
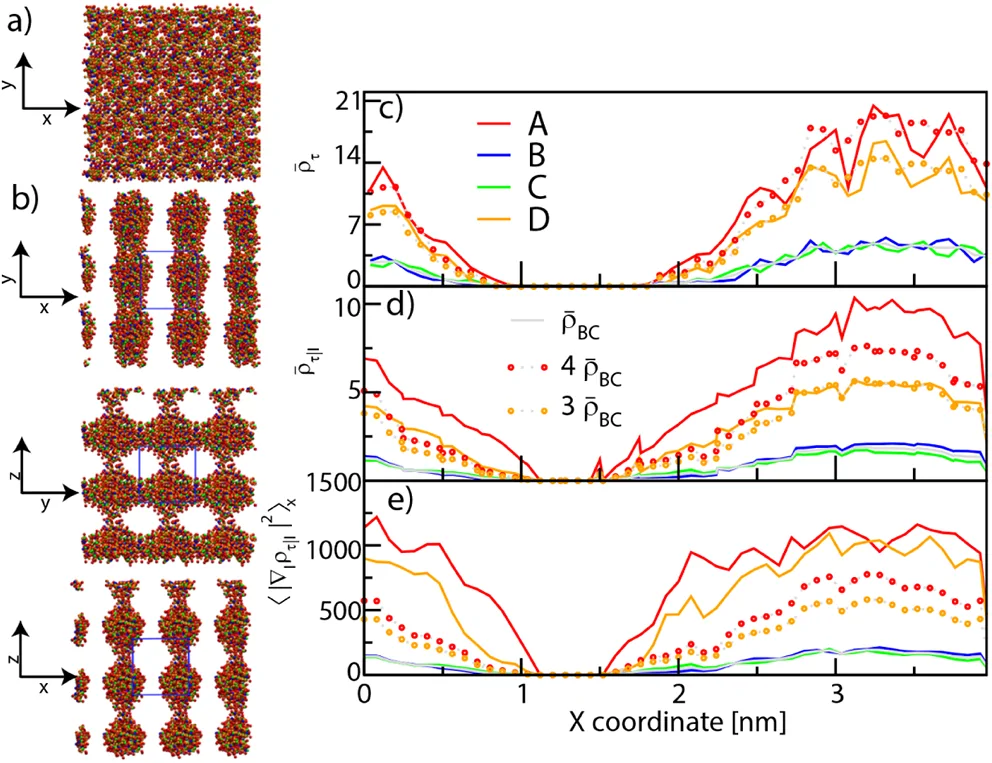
Analysis of simulated morphology. Panel a presents a visualization of the initial configuration, while panel b presents visualizations of the configuration after 6 ns of simulation. Panels c–e characterize the average density of τ = A, B, C, and D sites as a function of the x-coordinate in the simulation box. Specifically, panel c presents the average total density, ρ̅_τ_(*x*), of τ sites; panel d presents the average local density, ρ̅_τ|I_(*x*), of τ sites around each site, *I*, of type A; and panel e presents the average square gradient of the τ local density, ⟨|**∇**_I_ρ_τ|I_|^2^⟩*_x_*, around each site, *I*, of type A. Panels c, d, and e report densities and square gradients in units of c) nm^–3^; d) nm^–3^; and e) nm^–8^. The light gray curves in these panels average the corresponding curves for the B and C sites. The dotted red and orange curves in these panels scale this gray curve by factors of 4 and 3 in order to account for the multiplicity of A and B sites, respectively.

The remainder of Figure 8 characterizes the spatial distribution of sites transverse to the *x*-coordinate of the simulated box. The average partial densities, ρ̅_τ_(*x*), in Figure 8c indicate the vacuum region separating successive periodic sheets and largely follow the multiplicity of the various site types. The average local densities, ρ̅_τ|*I*_(*x*), in Figure 8d demonstrate that A sites preferentially associate with other A sites and tend to localize at the surface of the sheets, while B and C sites primarily localize in the interior. The average square gradients, ⟨|**∇**_*I*_ρ_τ|*I*_|^2^⟩_*x*_, around sites, *I*, of type A in Figure 8d demonstrate that A and D sites both localize in regions of relatively high density gradients.

We then employed BOCS to determine the simulated force functions from the net forces sampled in the LAMMPS simulation. This variational force-matching calculation represented the bond, angle, and dihedral force functions with 19 152 cubic spline polynomial functions, while representing the pair force, LD force, and SG coefficient functions with 5,957 cubic spline functions. Importantly, this calculation treated each of the 11 bonded potentials and each of the 42 nonbonded potentials as distinct. For example, we calculated all 10 distinct types of nonbonded pair force functions and did not assume that they were defined by the same function.

The dashed curves in Figure 7 present the 53 calculated force functions. BOCS determines the 26,348 potential parameters very accurately. Moreover, the force-matching calculation is quite robust with respect to statistical sampling. For instance, the SI demonstrates that the simulation rarely sampled configurations with 4.5 nm^–3^ ≤ ρ_*B*|*I*_, ρ_*C*|*I*_ ≤ 6 nm^–3^. Nevertheless, BOCS accurately determined the corresponding LD force and SG coefficient functions in this range.

### 4.2 Transferable 1-Site Water Model

Many previous studies have employed bottom-up approaches to parametrize pair potentials for one-site CG water models.^33,105–111^ In this final section, we investigate the utility of LD potentials for improving the accuracy and transferability of these models.^62,63,70^ Here we adopt SPC/E as a reasonably accurate all-atom (AA) water model.^94^ We consider CG models that represent each water molecule by a single site located at the molecular mass center.

The solid black curves in Figure 9a and b report the pressure–density equation of state and the mapped radial distribution function (rdf) obtained from a constant NPT AA simulation of bulk water at *T* = 300 K and an external pressure *P*_ext_ = 1 bar. The mapped rdf features a sharp first peak at *r* ≈ 0.276 nm that corresponds to hydrogen-bonding interactions, as well as a broader peak near *r* ≈ 0.458 nm that corresponds to the second solvation shell. The solid black curve in Figure 9c reports the interfacial density profile obtained from a constant NVT AA simulation of a liquid slab in coexistence with vapor at *T* = 300 K. The slab simulation forms a very sharp interface between a liquid phase with ρ_liq_ = 33.27 nm^–3^ and a vapor phase with ρ_vap_ = 0.00018 nm^–3^.

**Figure 9:**
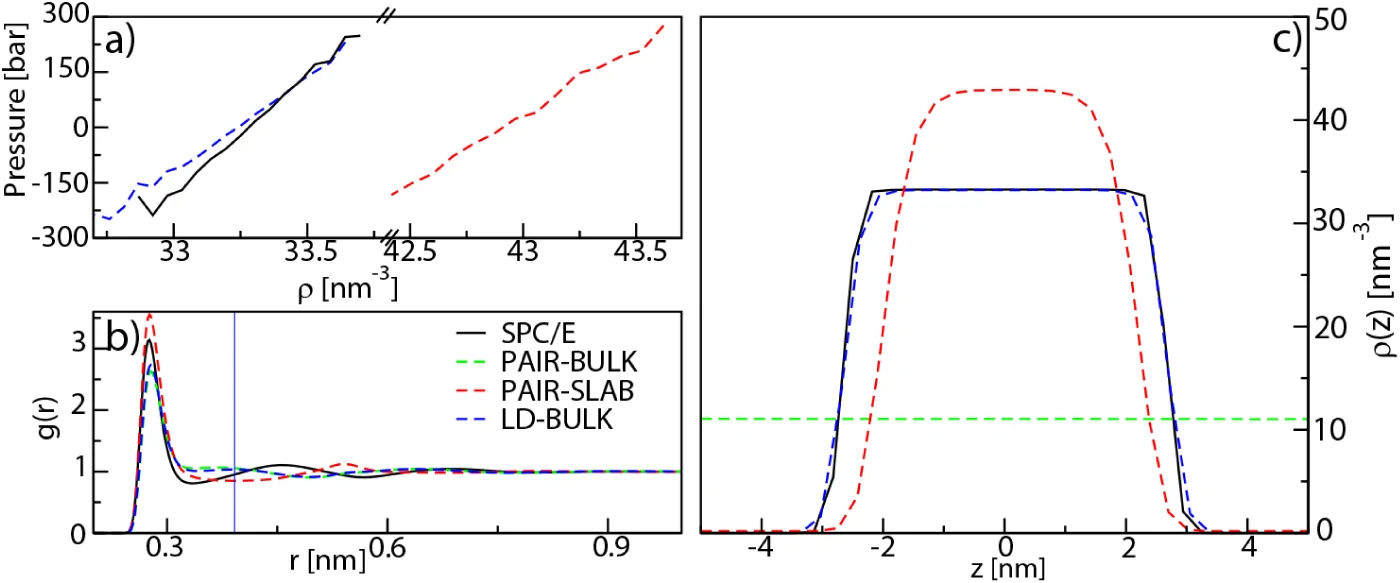
Characterization of SPC/E and CG water models. Panels a and b report the pressure–density equations of state and the simulated rdf’s, respectively, obtained from constant NPT bulk simulations at a temperature *T* = 300 K and an external pressure *P*_ext_ = 1 bar. Panel c reports the simulated density profile transverse to the z-coordinate from constant NVT slab simulations at a temperature *T* = 300 K. The solid black curves present results for the SPC/E model, while the dashed green, red, and blue curves present results for the PAIR-BULK, PAIR-SLAB, and LD-BULK models, respectively. Panel a does not include the pressure–density equation of state for the PAIR-BULK model because this model vaporized under the simulated conditions. Accordingly, the green curve in panel b reports the rdf obtained from a constant NVT simulation of the PAIR-BULK model performed at the average volume of the SPC/E constant NPT simulation. The vertical blue line in panel b indicates the local density length scale, *r*_c_ = 0.392 nm.

We employed BOCS to parametrize three different 1-site CG models via variational force-matching. The PAIR-BULK and PAIR-SLAB models described interactions with conventional pair potentials that were parametrized from the bulk and slab SPC/E simulations, respectively. Conversely, the LD-BULK model described interactions with both pair and LD potentials that were parametrized from the bulk water simulation. The LD-BULK model defined the local density by a Lucy weighting function with a length scale *r*_c_ = 0.392 nm that occurs early in the second solvation shell.

The solid curves in Figure 10a present the calculated pair potentials for the 3 CG models. Each pair potential features a very narrow minimum at *r* ≈ 0.28 nm that corresponds to hydrogen-bonding interactions, as well as a broader global minimum that occurs at *r* ≈ 0.41 nm near the second peak of the mapped rdf. The PAIR-BULK potential is very repulsive and also acts over a very short range. The PAIR-SLAB potential is not only more attractive, but also acts over a much longer distance range. Interestingly, the calculated LD-BULK pair potential is very similar to the PAIR-BULK pair potential at both short, *r* < 0.27 nm, and at long range, *r* > 0.6 nm. However, the LD-BULK pair potential is much more attractive over the intermediate regime, 0.27 nm < *r* < 0.6 nm. In particular, the global minima of the PAIR-SLAB and LD-BULK pair potentials are quite similar. Nevertheless, none of the minima in the calculated pair potentials are deeper than 1 *k*_*B*_ *T*.

**Figure 10:**
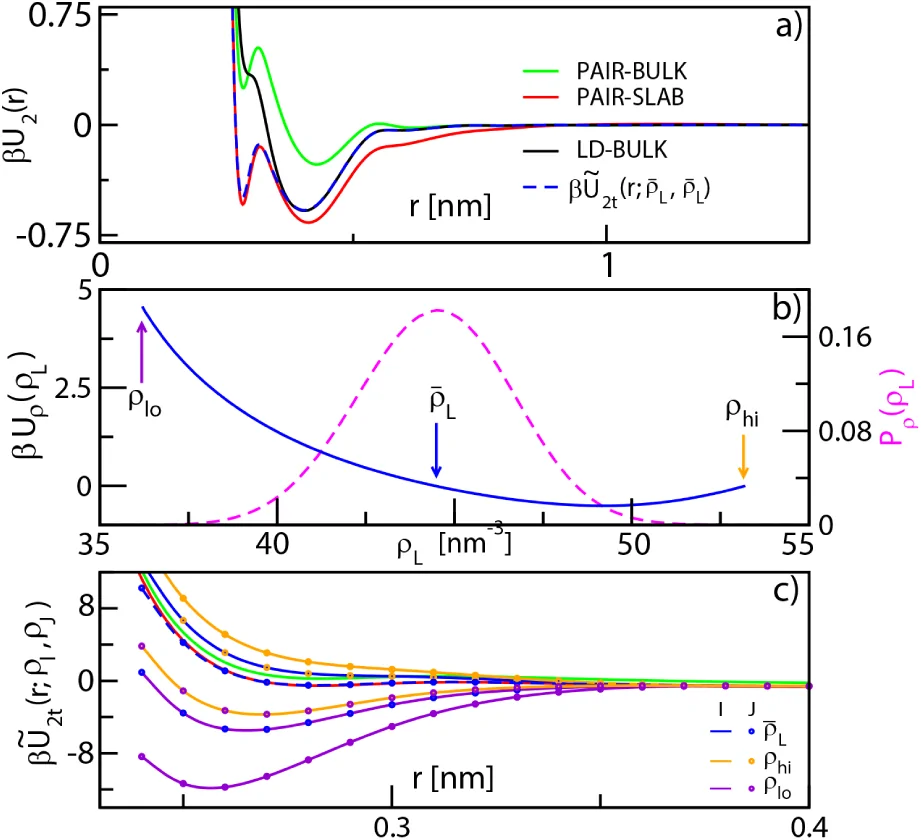
Interaction potentials for CG water models. The solid curves in panels a and b present the calculated pair potentials and the calculated LD potential for the LD-BULK model, respectively. The dashed magenta curve in panel b presents the local density distribution sampled by the bulk SPC/E simulation at the temperature *T* = 300 K and external pressure *P*_ext_ = 1 bar. The blue arrow in panel b indicates the mean, ρ̅_L_, of this distribution, while the purple and orange arrows indicate the minimum, ρ_lo_, and maximum, ρ_hi_, local densities sampled by this SPC/E simulation. Panel c analyzes the influence of the local densities, ρ_I_ and ρ_J_, of molecules *I* and *J*, respectively, upon the corresponding active pair potential, *Ũ*_2*t*_(*r;* ρ_I_, ρ_J_). In panel c, the purple, blue, and orange curves correspond to ρ_I_ = ρ_lo_, ρ̅_L_, and ρ_hi_, respectively, while the purple, blue, and orange symbols correspond to ρ_J_ = ρ_lo_, ρ̅_L_, and ρ_hi_, respectively. The green and red curves in panel c reproduce the PAIR-BULK and PAIR-SLAB pair potentials from panel a, respectively. The dashed blue curve in panel a presents the active pair potential for ρ_I_ = ρ_J_ = ρ̅_L_.

The solid blue curve in Figure 10b presents the calculated LD potential for the LD-BULK model. The dashed magenta curve in Figure 10b presents the distribution of local densities, ρ_L_, sampled by the bulk SPC/E simulation that was employed to parameterize the LD-BULK model. Because we include the self-term in defining the local density, the sampled local densities are significantly greater than the equilibrium bulk density of ρ_b_ = 33.3 nm^–3^. Moreover, because it is defined over such a short length-scale, the local density distribution is much broader than the simulated bulk density distribution. The calculated LD potential is attractive at most sampled local densities, including the average local density, ρ̅_L_. However, the LD potential becomes slightly repulsive at the highest local densities.

While the LD-BULK model generates pair-additive forces, these pair forces are modulated by the local environment of each pair. Specifically, the total force, *F*_2*t*_, between each pair, (*I*, *J*), is directed along the vector between the pair and may be expressed as

33 $$F_{2t}\left(R_{IJ},\rho_{I},\rho_{J}\right)=F_{2}\left(R_{IJ}\right)+\left[F_{\rho }\left(\rho_{I}\right)+F_{\rho }\left(\rho_{J}\right)\right]{\mathrm{w̅}}^{\prime }\left(R_{IJ}\right)$$

where *F*_2_(*r*) = −d*U*_2_(*r*)/d*r* is the pair force function, while *F*_ρ_(ρ) = −d*U*_ρ_(ρ)/dρ is the LD force function. Thus, the total pair force depends upon the pair distance, *R*_*IJ*_, as well as the local density, ρ_*I*_ and ρ_*J*_, of molecules *I* and *J*. Accordingly, we define an “active,” context-dependent pair potential, *Ũ*_2*t*_(*r*; ρ_*I*_, ρ_*J*_), for the LD-BULK model by integrating −*F*_2*t*_(*r*, ρ_*I*_, ρ_*J*_) with respect to *r*, while holding ρ_*I*_ and ρ_*J*_ constant. Because *w̅*(*r*) = 0 for *r* > *r*_c_, the local density only impacts *Ũ*_2*t*_(*r*; ρ_*I*_, ρ_*J*_) between pairs that are separated by *r* < *r*_c_. We note that the total potential of the LD-BULK model cannot be expressed in terms *Ũ*_2*t*_(*r*; ρ_*I*_, ρ_*J*_). Nevertheless, *Ũ*_2*t*_(*r*; ρ_*I*_, ρ_*J*_) provides useful qualitative insight into the impact of the environment upon pair interactions in the LD-BULK model.

Figure 10 illustrates the influence of the local environment upon the active pair potential, *Ũ*_2*t*_(*r*; ρ_*I*_, ρ_*J*_), for *r* < *r*_c_. The dashed blue curve in Figure 10a presents *Ũ*_2*t*_(*r*; ρ̅_L_, ρ̅_L_), which describes the interaction when both particles experience the average local density of the SPC/E model, i.e., ρ_*I*_ = ρ_*J*_ = ρ̅_L_. In this case, the active pair potential is very similar to the PAIR-SLAB potential in the first two solvation shells, but is very similar to the PAIR-BULK potential beyond the second solvation shell. Moreover, Figure 10c demonstrates that the active pair potential becomes even much more attractive as the local density decreases. This effect is especially pronounced in the first solvation shell as the hydrogen-bonding minimum not only deepens but also shifts to shorter distances with decreasing ρ_L_. In particular, if particle *I* experiences the average local density, ρ_*I*_ = ρ̅_L_, while particle *J* experiences the lowest local density, ρ_*J*_ = ρ_lo_, then the hydrogen-bonding minimum of the corresponding active pair potential is approximately −6*k*_*B*_ *T*. In this regime, the active potential is much more attractive than the PAIR-SLAB potential. Furthermore, if both particles are in a low density environment, ρ_*I*_ = ρ_*J*_ = ρ_lo_, then the hydrogen-bonding minimum deepens to approximately −12*k*_*B*_ *T*. Conversely, when both particles are in a high-density environment, ρ_*I*_ = ρ_*J*_ = ρ_hi_, then the hydrogen-bonding “minimum” is approximately +1*k*_*B*_ *T* and not even locally stable. In this case, the active potential is significantly more repulsive than the PAIR-BULK potential.

The dashed red and blue curves in Figure 9a and b report the results of constant NPT simulations with the PAIR-SLAB and LD-BULK models, respectively. Because the PAIR-BULK potential is almost entirely repulsive, this model dramatically overestimates the internal pressure and simply vaporizes at 1 bar of external pressure. Conversely, because the PAIR-SLAB potential is much more attractive, it stabilizes a condensed phase at 1 bar but overestimates the density and compressibility of the SPC/E model by 28.9% and 37.6%, respectively. As a consequence of this significantly enhanced density, the PAIR-SLAB model also describes the pair structure quite poorly and, in particular, significantly overestimates the first peak of the SPC/E rdf. The LD-BULK model reproduces the SPC/E equation of state much more accurately, underestimating the SPC/E density by only 0.1% and overestimating the SPC/E compressibility by 27.4%. Unfortunately, the LD-BULK model generally underestimates the order within the first solvation shell of the SPC/E model. For comparison, the solid green curve in Figure 9b reports the rdf obtained from constant NVT simulations of the PAIR-BULK model at the correct density. The inclusion of the LD potential slightly improves the agreement with the SPC/E rdf in the first solvation shell. Nevertheless, the similarity between the dashed blue and green curves indicates that the LD potential exerts a relatively small influence upon the local water structure.

The dashed, colored curves in Figure 9c report the density profiles obtained from constant NVT slab simulations with the three CG models. Because the PAIR-BULK model cannot stabilize the liquid phase at 1 bar pressure, this model forms a uniform, homogeneous phase. Conversely, the PAIR-SLAB model stabilizes liquid-vapor coexistence but significantly overestimates the density of the liquid phase, as already indicated in Figure 9b. Moreover, although it was parametrized from the SPC/E slab simulation, the PAIR-SLAB model badly overestimates the width of the liquid-vapor interface. In contrast, although it was parametrized from a bulk simulation, the LD-BULK model accurately reproduces the densities of the coexisting phases. The LD-BULK model also quite reasonably reproduces the width of the liquid-vapor interface.

Finally, we investigate the adsorption of each water model onto an attractive wall at *T* = 300 K. Specifically, we simulated each model in an extended rectangular box with finite length *L*_*z*_ = 25 nm in the *z*-direction, but periodic boundary conditions in the *x*- and *y*-directions. The dotted cyan curve in Figure 11b presents the external field, *V*(*z*), that models a perfectly smooth attractive wall at *z* = 0. This external field merges a rapidly varying repulsive LJ potential at short range with a slowly varying exponential attraction. This external field attains a minimum of approximately 1 *k*_*B*_ *T* at approximately *z* ≈ 0.3 nm and vanishes at approximately *z* ≈ 3 nm. The system is bounded on the right by a purely repulsive wall at *z* = 25 nm.

**Figure 11:**
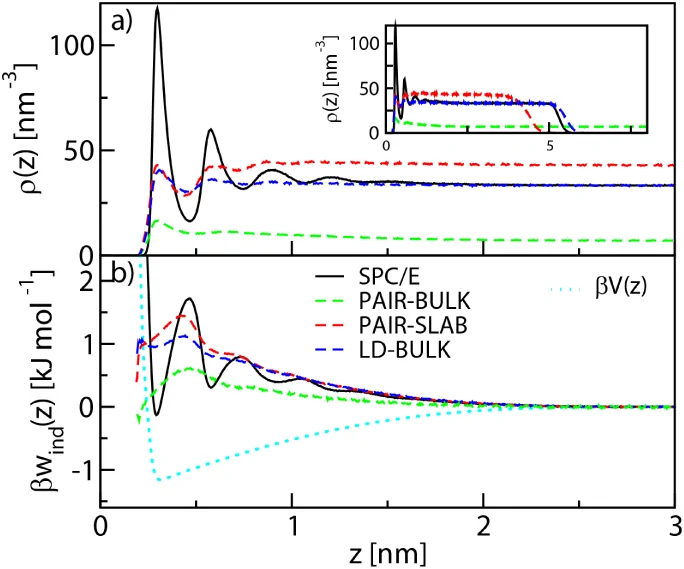
Analysis of SPC/E and CG water models in the presence of an attractive wall. Panel a presents density profiles describing adsorption at this wall, while the inset presents an expanded view of the same density profiles. Panel b presents the corresponding indirect contributions to the (scaled) pmf, β*w*_ind_(*z*) = −ln­[*e* ^+β*V*(*z*)^ρ­(*z*)/ρ_b_]. The solid black curves present results for the SPC/E model, while the dashed green, red, and blue curves present results for the PAIR-BULK, PAIR-SLAB, and LD-BULK models, respectively. The dotted cyan curve in panel b presents the (scaled) external field, β*V*(*z*).

Figure 11a demonstrates that the SPC/E model forms a very sharp, highly ordered interface with the attractive wall. The SPC/E density profile manifests four very pronounced interfacial layers before relaxing to the equilibrium liquid density by approximately 2 nm. At *z* ≈ 5 nm, the SPC/E model forms a liquid-vapor interface that is very similar to the interface reported in Figure 9c for the slab simulation.

It is instructive to consider the potential of mean force (pmf), *w*(*z*) ≡ −*k*_*B*_ *T* ln­[ρ­(*z*)/ρ_b_], for the SPC/E model in the presence of the external field. This pmf includes a direct contribution, *V*(*z*), from the external field and an indirect contribution from the effective potential, *w*_ind_(*z*) = −*k*_*B*_ *T* × ln­[*e* ^+β*V*(*z*)^ρ­(*z*)/ρ_b_], generated by intermolecular interactions. The black curve in Figure 11b demonstrates that this effective potential, *w*_ind_(*z*), is almost always positive but includes local minima corresponding to the maxima in the density profile.

Figure 11a demonstrates that the three CG models respond quite differently to the attractive walls. While the PAIR-BULK model adsorbs onto the attractive wall, the repulsive pair potential cannot stabilize a condensed phase. Consequently, the density profile for this model simply relaxes to that of its vapor density. Conversely, the PAIR-SLAB model stabilizes a liquid phase adjacent to an attractive wall. However, as already observed in Figure 9c, this model significantly overestimates the liquid phase density, as well as the interfacial width of the SPC/E model. Perhaps more surprisingly, this model also appears to dewet from the attractive wall as the density at contact is actually slightly less than the bulk liquid density.

The LD-BULK model most accurately reproduces the SPC/E density profile. As expected, the LD-BULK model accurately describes the liquid phase density and liquid-vapor interface. Moreover, the LD-BULK model clearly wets the attractive wall, as the corresponding density profile is largest at contact and gradually decays into the bulk liquid over approximately 2 nm. Nevertheless, the LD-BULK model significantly underestimates the ordering of the SPC/E model at the attractive wall. In particular, the LD-BULK density profile dramatically underestimates the first peak and also underestimates the magnitude of subsequent oscillations in the SPC/E density profile. Figure 11b demonstrates that the LD-BULK model qualitatively captures the features of the effective potential, *w*_ind_(*z*), due to intermolecular interactions. However, as perhaps an expected consequence of coarse-graining, the LD-BULK model smooths over the minima in *w*_ind_(*z*) that are responsible for the maximum in the density profile. This discrepancy may reflect the failure of the LD-BULK model to accurately describe the first solvation shell around each molecule. Additionally, Figure 10 suggests that the LD potential may destabilize the very dense interfacial layer that arises in the SPC/E model. It is possible that including an SG potential might allow the CG model to capture the detailed aspects of *w*_ind_(*z*) and, consequently, the SPC/E density profile, although this would likely require reparameterizing *r*_c_, *U*_2_, and *U*_ρ_. Alternatively, it is possible that employing the relative entropy approach^27,107^ to more accurately reproduce the mapped rdf may significantly improve the CG density profile.

We refer readers to ref 70 for a much more detailed analysis of LD potentials for 1-site water models. In particular, we employed variational force-matching to parametrize pair and LD potentials based upon a single bulk simulation of the TIP4P water model.^95^ Despite providing a rather poor description of the TIP4P RDF, this CG model reproduced cooperative hydrophobic phenomena quite accurately. Thus, LD potentials significantly improve the thermodynamic properties of CG models for bulk systems, their structural fidelity in describing inhomogeneous systems, and their transferability between bulk liquid and vapor phases as well as between bulk and interfacial environments.

## 5 Conclusions

Since its original release in 2017,^20^ BOCS has provided an accurate and robust software package for parametrizing bottom-up CG models of soft materials. In addition to implementing the force-matching variational principle for parametrizing molecular mechanics potentials,^21,32–34^ BOCS provided several unique capabilities. For instance, BOCS implemented a generalized Yvon–Born–Green theory for relating these potentials to structural correlation functions,^38,39,39–42^ a simple framework for improving their transferability across multiple systems,^43^ and a self-consistent pressure-matching method for accurately reproducing pressure–density equations of state.^45^

In this work, we have described a new release that dramatically expands the capabilities of BOCS for parametrizing LD and SG potentials via variational force-matching. This release is compatible with complex molecular topologies and implements a wide range of weighting functions for defining local density. In addition, we have described a significant extension of the PKG-BOCS package for simulating these LD and SG potentials in LAMMPS. This package implements flexible spline-based representations of these potentials as well as a library of functional forms that require fewer parameters. This package was included in the 4 July 2026 feature release of LAMMPS. Moreover, we are distributing Doxygen documentation and tutorials for using this software in the BOCS and LAMMPS.

In addition to describing this new software, we reported calculations illustrating its use for a series of relatively simple test models. While they did not provide insight into complex chemical systems, these calculations did provide physical insight into CG models with LD and SG potentials. For instance, they highlighted the influence of the local density weighting function, *w̅*, upon the simulated forces and equilibrium distribution. Similarly, by considering an isolated pair of particles, we gained insight into the complex distance-dependent forces generated by SG potentials. More importantly, these calculations provided a critical benchmark for this new software. Because we know the exact CG potential for these models by construction, these tests very precisely assess the accuracy and reliability of the variational force-matching calculation and the basis functions that we have implemented in BOCS. In particular, the third benchmark test demonstrated that BOCS can quantitatively determine more than 25 K parameters, defining over 50 distinct bonded, pair, LD, and SG interaction potentials for a CG model with a complex bonded topology. These results suggest that BOCS will provide an accurate and robust tool for parameterizing CG models of complex chemical systems in practice.

Moreover, we employed this software to investigate the accuracy and transferability of one-site CG water models that were parametrized via variational force-matching. These calculations explicitly illustrated the influence of LD potentials on modulating condensed-phase interactions as a function of the local environment. While they did not improve the bulk pair structure, LD potentials significantly improved the internal pressure of the CG model as well as its transferability between bulk and interfacial environments. In particular, the LD-BULK model quite accurately described both liquid-vapor coexistence and the liquid-vapor interface. However, this model underestimated the dramatic response of water to a smooth, attractive interface. This deficiency may stem from the repulsion generated by the LD potential in very dense environments. While this repulsion appears to improve the compressibility of the model,^70^ it may also destabilize the highly ordered interfacial layers. It would be very interesting to either supplement the potential with an SG term or parameterize a new LD potential in the presence of the attractive wall. However, these avenues are beyond the scope of this work.

The present work also indicates several additional directions for future study. For instance, the accuracy of LD potentials determined via force-matching depends quite strongly upon the length scale, *r*_c_, defining the local density. It would be very helpful to gain physical insight into predicting the optimal *r*_c_. Moreover, future studies should investigate the use of LD potentials in CG models with long-range electrostatics. Nevertheless, we hope that this new version of BOCS and the PKG-BOCS package in LAMMPS may prove to be useful for developing bottom-up CG models of complex soft materials.

## Supplementary Material



## Data Availability

The simulation results, analysis, and the exact LAMMPS and BOCS source codes described in this work are available from the Penn State DataCommons^112^ at 10.26208/PW48-SA96.^113^ Version 5.0 of the BOCS code that was used in the reported force-matching calculations may be accessed at https://github.com/noid-group/BOCS, along with corresponding documentation.^80^ The version of PKG-BOCS that was used in the reported simulations, along with rst/html style documentation for using it and interpreting the syntax, may be accessed at https://github.com/noid-group/lammps-BOCS2026. The final version of PKG-BOCS that has been implemented into LAMMPS may be accessed at https://github.com/lammps/lammps/pull/4737. The LAMMPS package is distributed under the GPL2 license, Copyright (2003) Sandia Corporation. Under the terms of Contract DE-AC04-94AL85000 with Sandia Corporation, the U.S. Government retains certain rights in this software. Available at https://www.lammps.org and https://github.com/lammps/lammps.

## 6 Appendices

### 6.1 Appendix 1: Basis Functions for Variational Force-Matching

The force field basis vectors associated with bonded and pairwise nonbonded potentials are easily derived. We define *U*_R0_ as the corresponding part of the total interaction potential:

34
$$U_{R0}\left(R\right)\equiv \underset{\zeta }{\sum }\underset{\lambda }{\sum }U_{\zeta }\left(\psi_{\zeta \lambda }\left(R\right)\right)$$

Here, ζ indicates a bonded or pairwise nonbonded interaction; *U*_ζ_ is the corresponding potential that is a function of a corresponding scalar variable, ψ_ζλ_(**R**), where λ indicates the particular instance of the interaction. For each of these potentials, we define a corresponding force function, *F*_ζ_(*x*) = −d*U*_ζ_(*x*)/d*x*, which we represent with a set of basis functions, *f*_ζ*d*_(*x*):

35
$$F_{\zeta }\left(x\right)=\underset{d}{\sum }\phi_{\zeta d}f_{\zeta d}\left(x\right)$$

where ϕ_ζ*d*_ are the corresponding force field coefficients. The total force from *U*_R0_ may then be expressed as follows:

36
$$F_{I;R0}\left(R\right)\equiv -\nabla_{I}U_{R0}\left(R\right)=\underset{\zeta }{\sum }\underset{d}{\sum }\phi_{\zeta d}G_{I;\zeta d}\left(R\right)$$

where the force field basis vector associated with *f*_ζ*d*_ is

37
$$G_{I;\zeta d}\left(R\right)\equiv \underset{\lambda }{\sum }f_{\zeta d}\left(\psi_{\zeta \lambda }\left(R\right)\right)\nabla_{I}\psi_{\zeta \lambda }\left(R\right)$$

The basis functions associated with LD and SG potentials are more complex. Here, we derive these basis vectors for the general case that the CG model treats multiple distinct types of CG sites. We first assign a type, τ_*I*_ ∈ {α, β, γ, ···}, for each particle *I* = 1, ..., *N*. This allows us to partition the set, $S_{I}$ , of particles that interact with site *I* via non-bonded interactions into contributions from each different type of site:

38
$$S_{I}=\underset{\tau }{\cup }S_{\tau |I}$$

where $S_{\tau |I}=\{J|\tau_{J}=\tau$ and *J* interacts with *I* via nonbonded potentials}. Note that $I\notin S_{I}$ . Moreover, the following are all equivalent: (1) $J\in S_{I}$ ; (2) $J\in S_{\tau_{J}|I}$ ; (3) $I\in S_{J}$ ; and (4) $I\in S_{\tau_{I}|J}$ . For each ordered pair of particle types, (*τ*_2_|*τ*_1_), we define a corresponding weighting function, ${\mathrm{w̅}}_{\tau_{2}|\tau_{1}}\left(r\right)$ . This allows us to define the local density, ρ_τ|*I*_, of τ sites around particle *I*:

39
$$\rho_{\tau |I}\equiv c_{\tau }\delta_{\tau \tau_{I}}+\underset{J\in S_{\tau |I}}{\sum }{\mathrm{w̅}}_{\tau |\tau_{I}}\left(R_{IJ}\right)$$

where *c*_τ_ is a configuration-independent constant that depends upon τ and accounts for any self-contribution to the local density since $I\notin S_{\tau |I}$ . Note that

40
$$\nabla_{I}\rho_{\tau |J}=\delta_{I,J}\underset{K\in S_{\tau |I}}{\sum }{\mathrm{w̅}}_{\tau |\tau_{I}}^{\prime }\left(R_{IK}\right){\mathrm{R̂}}_{IK}+\theta \left[I\in S_{\tau |J}\right]{\mathrm{w̅}}_{\tau_{I}|\tau_{J}}^{\prime }\left(R_{IJ}\right){\mathrm{R̂}}_{IJ}$$

where θ­[*C*] is an indicator function that equals 1 when the condition *C* is satisfied and otherwise vanishes. In particular, $\theta \left[I\in S_{\tau |J}\right]=\delta_{\tau_{I},\tau }\theta \left[I\in S_{J}\right]$ . (Note that $\theta \left[I\in S_{J}\right]=0$ if *I* = *J*.)

We define the LD potential:

41
$$U_{LD}\left(R\right)=\overset{N}{\underset{J=1}{\sum }}\underset{\tau }{\sum }U_{\tau |\tau_{J}}\left(\rho_{\tau |J}\right)$$

We define a force function, $F_{\tau_{2}|\tau_{1}}\left(\rho \right)=-dU_{\tau_{2}|\tau_{1}}\left(\rho \right)/d\rho$ , for each LD potential, $U_{\tau_{2}|\tau_{1}}$ . We represent $F_{\tau_{2}|\tau_{1}}\left(\rho \right)$ with a linear combination of basis functions:

42
$$F_{\tau_{2}|\tau_{1}}\left(\rho \right)=\underset{d}{\sum }\phi_{\tau_{2}|\tau_{1};d}f_{\tau_{2}|\tau_{1};d}\left(\rho \right)$$

This then defines the basis set for the LD potential:

43
$$F_{I;LD}\left(R\right)\equiv -\nabla_{I}U_{LD}\left(R\right)=\underset{\tau_{2}}{\sum }\underset{\tau_{1}}{\sum }\underset{d}{\sum }\phi_{\tau_{2}|\tau_{1};d}G_{I;\tau_{2}|\tau_{1}d}\left(R\right)$$

where the force field basis vector, $G_{I;\tau_{2}|\tau_{1}d}\left(R\right)$ , associated with $U_{\tau_{2}|\tau_{1}}$ may be expressed as

44
$$\begin{array}{ll} G_{I;\tau_{2}|\tau_{1}d}\left(R\right)= & \delta_{\alpha \tau_{1}}f_{\tau_{2}|\alpha ;d}\left(\rho_{\tau_{2}|I}\right)\underset{J\in S_{\tau_{2}|I}}{\sum }{\mathrm{w̅}}_{\tau_{2}|\alpha }^{\prime }\left(R_{IJ}\right){\mathrm{R̂}}_{IJ}\\ & +\delta_{\alpha \tau_{2}}\underset{J\in S_{\tau_{1}|I}}{\sum }f_{\alpha |\tau_{1};d}\left(\rho_{\alpha |J}\right){\mathrm{w̅}}_{\alpha |\tau_{1}}^{\prime }\left(R_{IJ}\right){\mathrm{R̂}}_{IJ} \end{array}$$

where we have set α ≡ τ_*I*_ to slightly simplify the expression.

In order to define the basis vectors for the SG potential, we first define **A**_*τ*|*J*_ = ∇_*J*_ρ_τ|*J*_ = (*A*_*τ*|*Jj*_) with *A*_τ|*Jj*_ = ∇_*Jj*_ρτ_|_ *_J_* = ∂ρ_τ|*J*_/∂*R*_*Jj*_, where the lower case letters indicate Cartesian directions, *i*, *j* = 1, 2, 3. We also need to evaluate

45
$$\frac{\partial }{\partial R_{Ii}}A_{\tau |Jj}=\underset{K\in S_{\tau |J}}{\sum }\delta_{I|JK}L_{\tau K|Jij}$$

where δ_*I*|*JK*_ = δ_*IJ*_ – δ_*IK*_ and we have also introduced the matrix, *L*_τ*K|J*_ = (*L*_τ*K*|*Jij*_), with elements

46
$$L_{\tau K|Jij}=f_{\tau |J}\left(R_{JK}\right)\delta_{ij}+g_{\tau |J}\left(R_{JK}\right){\mathrm{R̂}}_{JKi}{\mathrm{R̂}}_{JKj}$$

where

47
$$f_{\tau |J}\left(r\right)=r^{-1}{\mathrm{w̅}}_{\tau |\tau_{J}}^{\prime }\left(r\right)$$

48
$$g_{\tau |J}\left(r\right)={\mathrm{w̅}}_{\tau |\tau_{J}}^{\prime \prime }\left(r\right)-f_{\tau |J}\left(r\right)$$

and **R̂**_*JKi*_ is the *i* ^th^ Cartesian component of the unit vector, **R̂**_*JK*_. This matrix allows us to express

49
$$\frac{\partial }{\partial R_{I}}{|\nabla_{J}\rho_{\tau |J}|}^{2}=\frac{\partial }{\partial R_{I}}A_{\tau |J}^{2}=2\underset{K\in S_{\tau |J}}{\sum }\delta_{I|JK}B_{\tau K|J}$$

where

50
$$B_{\tau K|J}\equiv L_{\tau K|J}A_{\tau |J}=f_{\tau |J}\left(R_{JK}\right)A_{\tau |J}+g_{\tau |J}\left(R_{JK}\right)\left({\mathrm{R̂}}_{JK}\cdot A_{\tau |J}\right){\mathrm{R̂}}_{JK}$$

We now define the more general SG potential:

51
$$U_{SG}\left(R\right)=\overset{N}{\underset{J=1}{\sum }}\underset{\tau }{\sum }U_{\nabla \tau |\tau_{J}}\left(\rho_{\tau |J}\right)A_{\tau |J}^{2}$$

We represent the coefficient function, $U_{\nabla \tau_{2}|\tau_{1}}$ , with a simple basis set, which then determines a corresponding representation of $F_{\nabla \tau_{2}|\tau_{1}}\left(\rho \right)=-dU_{\nabla \tau_{2}|\tau_{1}}\left(\rho \right)/d\rho$ :

52
$$U_{\nabla \tau_{2}|\tau_{1}}\left(\rho \right)=\underset{d}{\sum }\phi_{\nabla \tau_{2}|\tau_{1};d}f_{\nabla \tau_{2}|\tau_{1};d}\left(\rho \right)$$

53
$$F_{\nabla \tau_{2}|\tau_{1}}\left(\rho \right)=-\underset{d}{\sum }\phi_{\nabla \tau_{2}|\tau_{1};d}f_{\nabla \tau_{2}|\tau_{1};d}^{\prime }\left(\rho \right)$$

This then determines the basis set for the SG potential:

54
$$F_{I;SG}\left(R\right)\equiv -\nabla_{I}U_{SG}\left(R\right)=\underset{\tau_{2}}{\sum }\underset{\tau_{1}}{\sum }\underset{d}{\sum }\phi_{\nabla \tau_{2}|\tau_{1};d}G_{I;\nabla \tau_{2}|\tau_{1};d}\left(R\right)$$

where

55
$$G_{I;\nabla \tau_{2}|\tau_{1};d}\left(R\right)=G_{I;\nabla \tau_{2}|\tau_{1};d}^{\left(1\right)}+G_{I;\nabla \tau_{2}|\tau_{1};d}^{\left(2\right)}$$

and

56
$$G_{I;\nabla \tau_{2}|\tau_{1};d}^{\left(1\right)}=-\delta_{\alpha \tau_{1}}\underset{J\in S_{\tau_{2}|I}}{\sum }\left\{f_{\nabla \tau_{2}|\alpha ;d}^{\prime }\left(\rho_{\tau_{2}|I}\right)A_{\tau_{2}|I}^{2}{\mathrm{w̅}}_{\tau_{2}|\alpha }^{\prime }\left(R_{IJ}\right){\mathrm{R̂}}_{IJ}+2f_{\nabla \tau_{2}|\alpha ;d}\left(\rho_{\tau_{2}|I}\right)B_{\tau_{2}J|I}\right\}$$

57
$$G_{I;\nabla \tau_{2}|\tau_{1};d}^{\left(2\right)}=-\delta_{\alpha \tau_{2}}\underset{J\in S_{\tau_{1}|I}}{\sum }\left\{f_{\nabla \alpha |\tau_{1};d}^{\prime }\left(\rho_{\alpha |J}\right)A_{\alpha |J}^{2}{\mathrm{w̅}}_{\alpha |\tau_{1}}^{\prime }\left(R_{IJ}\right){\mathrm{R̂}}_{IJ}-2f_{\nabla \alpha |\tau_{1};d}\left(\rho_{\alpha |J}\right)B_{\alpha I|J}\right\}$$

### 6.2 Appendix 2: 2 Particle SG Invariance

The definition of the SG potential $U_{\mathrm{SG}}\left(R\right)\equiv$ $\overset{N}{\underset{I=1}{\sum }}U_{\nabla }\left(\rho_{I}\right){|\nabla_{I}\rho_{I}|}^{2}$ in eq 8 implies that the forces and the canonical configuration distribution are unchanged when the SG coefficient function, *U*_**∇**_(ρ_*I*_), is replaced with the generalized coefficient *Ũ*_**∇**_(ρ_*I*_, **∇**_*I*_ρ_*I*_) = *U*_**∇**_(ρ_*I*_) + *c*|**∇**_*I*_ρ_*I*_|^–2^. Section 4.1.2 considers a system of *N* = 2 particles interacting via SG potential, for which ρ_*I*_ = *w̅*(*R*_12_) and |**∇**_*I*_ρ_*I*_|^2^ = |*w̅*′(*R*_12_)|^2^. In this case, ρ_*I*_ and *R*_12_ are in 1-to-1 correspondence for 0 < *R*_12_ < *r*_*c*_, which implies that |**∇**_*I*_ρ_*I*_|^–2^ is a function of ρ_*I*_. Thus, for *N* = 2, the generalized coefficient may be expressed as a function of only the local density, i.e., *Ũ*_**∇**_ = *Ũ*_**∇**_(ρ_*I*_). Below, we explicitly derive this invariance for *N* = 2.

In the case of *N* = 2, the total potential can be expressed in terms of the effective pair potential, *Ũ*_2_, given by eq 31 that corresponds to the effective pair force, *F̃*_2_, given by eq 32. We now consider replacing *U*_**∇**_(ρ_*I*_) with *Ũ*_**∇**_(ρ_*I*_) ≡ *U*_**∇**_(ρ_*I*_) + *y*(ρ_*I*_), where *y*(ρ_*I*_) is an unknown function. The equilibrium distribution will remain unchanged as long as the effective pair force remains unchanged. By substituting *Ũ*_**∇**_(ρ_*I*_) into eq 32, we see that this invariance is achieved when *y* satisfies the following differential equation:

58
$${\mathrm{w̅}}^{\prime }\left(r\right)\left[y^{\prime }\left(\rho_{r}\right){|{\mathrm{w̅}}^{\prime }\left(r\right)|}^{2}+2y\left(\rho_{r}\right){\mathrm{w̅}}^{\prime \prime }\left(r\right)\right]=0$$

where ρ_*r*_ = *w̅*(*r*). We consider the distance range over which *w̅*′(*r*) < 0 and assume that *y*(ρ_*r*_) > 0 over this range; eq 58 is then equivalent to

59
$$0=\frac{y^{\prime }\left(\rho_{r}\right)}{y\left(\rho_{r}\right)}{\mathrm{w̅}}^{\prime }\left(r\right)+2\frac{{\mathrm{w̅}}^{\prime \prime }\left(r\right)}{{\mathrm{w̅}}^{\prime }\left(r\right)}=\frac{d}{dr}\left\{\ln\left[y\left(\rho_{r}\right){|{\mathrm{w̅}}^{\prime }\left(r\right)|}^{2}\right]\right\}$$

which is satisfied when *y*(ρ_*r*_) = *c*|*w̅*′(*r*)|^–2^ or, equivalently,

60
$$y\left(\rho \right)=c{|{\mathrm{w̅}}^{\prime }\left(r_{\rho }\right)|}^{-2}$$

where *r*_ρ_ is the unique distance for which *w̅*(*r*_ρ_) = ρ. Consequently, there exists a one-parameter family of SG coefficient functions *Ũ*_**∇**_(ρ; *c*) = *U*_**∇**_(ρ) + *c*|*w̅*′(*r*_ρ_)|^–2^ that generate the same forces on a pair of isolated particles.

If we had represented *U*_**∇**_(ρ_L_) with a flexible spline basis set, then this invariance would have complicated our assessment of the SG test in Section 4.1.2. Specifically, this invariance would imply that a family of equivalent SG coefficient functions ${Ũ}_{\nabla }^{\mathrm{FM}}\left(\rho ;c\right)=U_{\nabla }^{\mathrm{FM}}\left(\rho \right)+c{|{\mathrm{w̅}}^{\prime }\left(r_{\rho }\right)|}^{-2}$ all minimized the force-matching variational functional, χ^2^[*U*]. However, we avoided these complications by representing *U*_**∇**_ with a less flexible basis set that was not compatible with *y*(ρ) = *c*|*w̅*′(*r*_ρ_)|^–2^.

## Notes

Additional details of the local density weighting functions and force-matching calculations, as well as additional analysis of the molecular test system (PDF)
