# Supplementary material for: Progress toward a Better BOCS: Systematic Coarse-Graining with Local Density Potentials
Source: J Chem Theory Comput. 2026 Oct 12;22(20):10616–35. doi: 10.1021/acs.jctc.6c01228 (PMC13718548; doi:10.1021/acs.jctc.6c01228)
Supplement: Supplementary file 1 [file ct6c01228_si_001.pdf]

Supporting Information for Progress toward a better BOCS: Systematic coarse-graining with local density potentials

Maria C. Lesniewski, Michael R. DeLyser, and W. G. Noid*

Department of Chemistry, The Pennsylvania State University, University Park, Pennsylvania 16802, USA

E-mail: wgn1@psu.edu

Abstract

This document provides supporting information (SI) for the manuscript "Progress toward a better BOCS: Systematic coarse-graining with local density potentials." Section 1 defines the local density weighting functions that have been implemented into BOCS and PKG-BOCS. Section 2 provides computational details for the three benchmark tests described in the main text. Section 3 details the hyperparameters employed in the force-matching calculations for these benchmarks. Section 4 provides additional analysis of the third test reported in the manuscript.

1 Weighting functions

Equation (6) of the manuscript defines the local density, ρ_I, in terms of a normalized weighting function,

$$\overline{w}(r) = \frac{w(r)}{[w]}, \tag{1}$$

where $w(r)$, is a non-increasing function that is 1 when $r = 0$ and vanishes when $r > r_c$, while $[w]$ is the normalization

$$[w] = \int_0^{r_c} 4\pi r^2 w(r) dr. \tag{2}$$

Below we explicitly define the local density weighting functions that we have implemented into BOCS version 5.0 and the PKG-BOCS package in LAMMPS. Table S1 summarizes this information. While each weighting function vanishes beyond the local density length-scale, r_c, the Shell, smooth, and sphere weighting functions also incorporate a second hyperparameter, r_0. These latter three weighting functions have a constant value of 1 over the interval $0 \leq r \leq r_0$ and decrease to 0 over the interval $r_0 \leq r \leq r_c$. In Fig. 2 of the main text we set $r_0 = r_c/2$, while in the reported test calculations we set $r_0 = 0$.

Table S1: Summary of weighting functions that we have implemented in BOCS and the PKG-BOCS package in LAMMPS.

Indicator	hyperparameters	$w(r)$	$[w]$
DPD	r_c	Eq. (3)	Eq. (4)
Lucy	r_c	Eq. (5)	Eq. (6)
Shell	r_0, r_c	Eq. (7)	Eq. (13)
smooth	r_0, r_c	Eq. (14)	Eq. (22)
sphere	r_0, r_c	Eq. (25)	Eq. (30)

1.1 DPD weighting function

Dissipative particle dynamics (DPD) models[1–3] often employ the weighting function

$$w(r) = (1 - r/r_c)^2 \theta(1 - r/r_c), \tag{3}$$

where the Heaviside function $\theta(x) = 0$ for $x < 0$ and $= 1$ for $x > 0$. The corresponding normalization is

$$[w] = 2\pi r_c^3/15. \tag{4}$$

The first derivative of the DPD weighting function is continuous everywhere, while the second derivative is discontinuous at $r = r_\mathrm{c}$.

1.2 Lucy weighting function

Local density (LD) potentials that are parameterized via force-matching[4–8] often define the local density with the Lucy function,[9,10]

$$w(r) = (1 - r/r_c)^3(1 + 3r/r_c)\theta(1 - r/r_\mathrm{c}). \tag{5}$$

The corresponding normalization is

$$[w] = 16\pi r_c^3/105. \tag{6}$$

The first and second derivatives of the Lucy function are continuous for all $r > 0$.

1.3 Shell weighting function

Sanyal and Shell defined the local density with the following weighting function[11,12]

$$w(r) = \begin{cases} 1 & r \le r_0 \\ d^{-1}(c_0 + c_2 r^2 + c_4 r^4 + c_6 r^6) & r_0 \le r \le r_c \\ 0 & r \ge r_c \end{cases} \tag{7}$$

where

$$d = (1 - r_0^2/r_c^2)^3, \tag{8}$$

$$c_0 = (1 - 3r_0^2/r_c^2), \tag{9}$$

$$c_2 = 6r_0^2/r_c^4, \tag{10}$$

$$c_4 = -3(1 + r_0^2/r_c^2)/r_c^4, \tag{11}$$

$$c_6 = 2/r_c^6 \tag{12}$$

The corresponding normalization is

$$[w] = 4\pi \left\{ \frac{1}{3} r_0^3 + \frac{1}{d} \left[\frac{1}{3} c_0 (r_c^3 - r_0^3) + \frac{1}{5} c_2 (r_c^5 - r_0^5) + \frac{1}{7} c_4 (r_c^7 - r_0^7) + \frac{1}{9} c_6 (r_c^9 - r_0^9) \right] \right\}. \tag{13}$$

The first derivative of the Shell weighting function is continuous everywhere, while the second derivative is discontinuous at r_0 and r_c.

1.4 Smooth weighting function

We have introduced a new weighting function that is also constant over the interior interval $0 < r < r_0$ with higher order continuity:

$$w(r) = \begin{cases} 1 & r \leq r_0 \\ d^{-1} \sum_{i=0}^{5} c_i r^i & r_0 \leq r \leq r_c \\ 0 & r \geq r_c \end{cases} \tag{14}$$

where the coefficients are

$$d = (r_0^5 - r_c^5)/120 - (r_0^3 - r_c^3)r_0 r_c/24 + (r_0 - r_c)r_0^2 r_c^2/12, \tag{15}$$

$$c_0 = -r_c^5/120 + r_0 r_c^4/24 - r_0^2 r_c^3/12, \tag{16}$$

$$c_1 = r_0^2 r_c^2/4, \tag{17}$$

$$c_2 = -r_0 r_c(r_0 + r_c)/4, \tag{18}$$

$$c_3 = (r_0^2 + 4r_0 r_c + r_c^2)/12, \tag{19}$$

$$c_4 = -(r_0 + r_c)/8, \tag{20}$$

$$c_5 = 1/20 \tag{21}$$

The normalization is

$$[w] = 4\pi \left[r_0^3/3 + d^{-1} \sum_{i=0}^{5} \frac{c_i}{i+3}(r_c^{i+3} - r_0^{i+3}) \right] \tag{22}$$

The second derivative of the smooth indicator function is continuous everywhere and, in particular, vanishes for both $r \le r_0$ and for $r \ge r_c$.

1.5 Sphere Indicator

We have also implemented a weighting function that describes the overlap between two spheres. Consider a large sphere of radius X and a small sphere of radius x, whose centers are separated by a distance r. We define

$$X = (r_0 + r_c)/2 \tag{23}$$

$$x = (r_c - r_0)/2. \tag{24}$$

such that $r_0 = X - x$ and $r_\mathrm{c} = X + x$. The fraction of the small sphere that overlaps with the large sphere is

$$w(r) = \begin{cases} 1 & r \leq r_0 \\ c_3 r^3 + c_1 r + c_0 + c_{-1}/r & r_0 \leq r \leq r_c \\ 0 & r_\mathrm{c} \leq r \end{cases} \tag{25}$$

where

$$c_{-1} = 3(X^4 + x^4)/(16x^3) + 3X^2/(8x) \tag{26}$$

$$c_0 = (X^3 + x^3)/(2x^3) \tag{27}$$

$$c_1 = -3(X^2 + x^2)/(8x^3) \tag{28}$$

$$c_3 = 1/(16x^3) \tag{29}$$

The normalization is

$$[w] = 4\pi \left[\frac{1}{3} r_0^3 + \sum_{i \in \{-1,0,1,3\}} \left(\frac{c_i}{i+3} \right) (r_c^{i+3} - r_0^{i+3}) \right] \tag{30}$$

The first derivative of the sphere weighting function is continuous everywhere, while its second derivative is discontinuous at both r_0 and r_c.

2 Computational details of benchmark test studies

2.1 Test 1: 400 molecules with linear LD potential

The first test considered $N = 400$ equivalent spherical particles that interacted via the potential $U_\mathrm{R}(\mathbf{R}) = \sum_I^N U_\rho(\rho_I)$, where $U_\rho(\rho_\mathrm{L}) = -k\rho_\mathrm{L}$ with $k = 1$ kJ $\cdot$ mol^{-1} $\cdot$ nm^3. We defined the local density, ρ_I, around each molecule, I, with a weighting function, $\overline{w}(r)$, that

vanished for $r \geq r_c = 1.0$ nm. We considered 5 different variations on this test that employed the 5 weighting functions, $\overline{w}(r)$, described above and in Section 3.1.3 of the main text.

We created the initial configuration by randomly placing the 400 particles within a sphere of radius 1.5 nm at the center of a cubic box with sides of length $L = 10$ nm. We simulated this initial condition for 10 ns, while using a 1 fs time step to integrate the equations of motion and a neighbor list with $r_{\text{list}} = 2.0$ nm. This simulation employed the default fix nvt settings to sample the canonical ensemble at $T = 270$ K via a Nosé-Hoover chain thermostat[13–15] with a chain of length $n = 3$ and relaxation time scale of 100 fs. We sampled thermodynamic and trajectory information every 500 time steps.

We employed two different basis sets for the variational force-matching calculation. The first basis set employed only LD force functions, $F_\rho(\rho_L)$. In this case, we represented $F_\rho(\rho_L)$ with 4th order (cubic) splines, while employing a knot spacing and domain tailored to the local density range sampled by the LAMMPS simulation. The second basis set employed only pair force functions, $F_2(r)$. We represented $F_2(r)$ over the interval $0.001 \text{ nm} \leq r \leq 1.5$ nm with 4th order (cubic) splines, while employing a knot spacing $\delta r = 0.01$ nm.

2.2 Test 2: 2 particles with LD or SG potentials

The second test considered $N = 2$ equivalent spherical particles and two distinct cases. In the first LD case the particles interacted via the potential $U_R(\mathbf{R}) = U_{LD}(\mathbf{R}) = \sum_I^N U_\rho(\rho_I)$, where $U_\rho(\rho_L) = A\rho_L^2 + B\rho_L + C$ with $A = 2 \text{ kcal} \cdot \text{Å}^6 \cdot \text{mol}^{-1}$, $B = -4.4 \text{ kcal} \cdot \text{Å}^3 \cdot \text{mol}^{-1}$, and $C = 2.42 \text{ kcal} \cdot \text{mol}^{-1}$. In the second SG case the particles interacted via the potential $U_R(\mathbf{R}) = U_{SG}(\mathbf{R}) = \sum_I^N U_{\boldsymbol{\nabla}}(\rho_I)|\boldsymbol{\nabla}_I \rho_I|^2$, where $U_{\boldsymbol{\nabla}}(\rho_L) = \tilde{A}\rho_L + \tilde{B}$ with $\tilde{A} = 32$ kcal $\cdot \text{Å}^{11} \cdot \text{mol}^{-1}$ and $\tilde{B} = -64 \text{ kcal} \cdot \text{Å}^8 \cdot \text{mol}^{-1}$. We considered 5 variants of each case that defined the local density according to the 5 weighting functions, $\overline{w}(r)$, described above and in Section 3.1.3 of the main text. The LD and SG cases defined the local density length scale by $r_c = 1.2$ Å and 2.0 Å, respectively.

In each case we initially placed the two particles 1 Å away from each other. We then

simulated them in a cubic box with sides of fixed length $L = 7$ Å for a total of 6.3×10^9 time steps, while integrating the equations of motion with a 1 fs timestep. These simulations employed a Langevin[16,17] thermostat with a 50 fs damping factor to sample the canonical ensemble at $T = 270$ K. The simulations for the LD and SG cases employed neighbor lists with $r_{\text{list}} = 2.4$ Å and 4 Å, respectively. In both cases, we checked at every time step whether the neighbor list needed to be rebuilt. We sampled every 500th configuration for analysis. We determined the conservative forces by postprocessing these sampled configurations with the LAMMPS fix nvt command.

In both LD and SG cases, we performed two independent force-matching calculations with different basis sets. Importantly, the basis functions employed in these force-matching calculations do not perfectly represent either the simulated force functions or the corresponding effective pair force functions. Consequently, we did not assess the error in the force-matching calculations by comparing the exact and calculated functions at each knot point.

1. The first force-matching calculation employed basis functions of the local density, ρ_{L}. In the LD case we represented the LD force function, $F_\rho(\rho_{\text{L}})$, with 4th order (cubic) spline functions. These calculations employed approximately 10 evenly spaced knots over the range of simulated local densities. In the SG case we represented the SG coefficient function, $U_{\boldsymbol{\nabla}}(\rho_{\text{L}})$, with a single 2nd order (linear) spline function. We quantified the error in the force-matching calculations for the LD case by directly comparing the simulated LD force function with the calculated LD force function. We quantified the error in the force-matching calculations for the SG case by comparing the net forces that are generated by the simulated and calculated SG potentials. We computed these net forces by post-processing the sampled configurations with the corresponding SG potentials.

2. The second force-matching calculation employed basis functions of the pair distance. In the LD case, we represented the pair force function, $F_2(r)$, by linear spline functions

with knots spaced every $\delta r = 0.0001$ nm over the interval 0.0 nm $\leq r \leq 0.2$ nm. In the SG case, we represented $F_2(r)$ by cubic spline functions with knots spaced every $\delta r = 0.0005$ nm over the interval 0.0 nm $\leq r \leq 0.2$ nm. The only exception occurred for the Lucy function. In this case we represented $F_2(r)$ by cubic spline functions with knots spaced every $\delta r = 0.002$ nm over the interval 0.0 nm $\leq r \leq 0.2$ nm. We quantified the error in these force-matching calculations by averaging the difference between the standard function and the calculated function within each knot interval. Specifically, we computed this difference at 300 evenly spaced points within each knot interval and divided the sum of these differences by 300.

The simulated ensembles for this second test also provided an independent validation of the LD and SG potentials implemented in LAMMPS. For the present case of $N = 2$ particles, the total potential may be re-expressed in terms of an effective pair potential, $U(\mathbf{R}) = \widetilde{U}_2(R_{12})$, where R_{12} is the distance between the two particles. Consequently, the canonical probability (density) for finding the two particles separated by a distance r is $P_{\mathrm{r}}(r) \propto r^2 \exp[-\beta \widetilde{U}_2(r)]$. In particular, the regime $r > r_{\mathrm{c}}$ corresponds to free particles for which $P_{\mathrm{r}}(r) \propto r^2$. In the present work we determined the constant of proportionality, C, by fitting the sampled distribution of pair distances to $P_{\mathrm{r}}(r) = Cr^2 \exp[-\beta \widetilde{U}_2(r)]$. This canonical pair distribution then determines the canonical distribution of local densities, $P_\rho(\rho_{\mathrm{L}})$, which may be decomposed :

$$P_\rho(\rho_{\mathrm{L}}) = P_{\mathrm{b}}(\rho_{\mathrm{L}}) + P_{\mathrm{f}} \delta(\rho_{\mathrm{L}}) \tag{31}$$

where P_{b} and $P_{\mathrm{f}} = 1 - P_{\mathrm{b}}$ correspond to contributions from bound and free configurations, respectively. Additionally, in the present case of $N = 2$ particles, the local density is simply $\rho_{\mathrm{L}} = \rho_1 = \rho_2 = \overline{w}(R_{12})$. Because $\overline{w}$ monotonically decreases over the interval $0 \leq r \leq r_{\mathrm{c}}$, there exists a one-to-one relationship between the interparticle distance, $0 \leq R_{12} \leq r_{\mathrm{c}}$, and the local density, $0 \leq \rho_{\mathrm{L}} \leq \rho_{\mathrm{max}}$. This allows us to determine the bound contribution to the

canonical probability distribution:

$$P_{\mathrm{b}}(\rho_{\mathrm{L}}) = P_{\mathrm{r}}(r) \left. \left| \frac{\mathrm{d}\overline{w}}{\mathrm{d}r} \right|^{-1} \right|_{r=R_{\rho_{\mathrm{L}}}}, \tag{32}$$

where $R_{\rho_{\mathrm{L}}}$ is the distance corresponding to ρ_{L}, i.e., $\rho_{\mathrm{L}} = \overline{w}(R_{\rho_{\mathrm{L}}})$.

2.3 Test 3: 125 molecules with LD and SG potentials

The third test considered a system with 125 molecules and 4 different types of CG sites, $\tau =$ A, B, C, or D. Each molecule consisted of 9 sites with a branched topology. The interaction potential included both bonded and nonbonded contributions, $U_{\mathrm{R}}(\mathbf{R}) = U_{\mathrm{b}}(\mathbf{R}) + U_{\mathrm{nb}}(\mathbf{R})$. The bonded potential, U_{b}, treated 11 distinct types of interactions, including 4 bond potentials, 5 angle potentials, and 2 dihedral potentials. The nonbonded potential, U_{nb}, treated 42 distinct types of interactions, including 10 pair potentials, 16 LD potentials, and 16 SG potentials. The nonbonded potential governed both intermolecular interactions and also intramolecular interactions between sites that were separated by 4 or more bonds, i.e., we excluded nonbonded interactions between intramolecular 1-2, 1-3, and 1-4 pairs. We represented all 42 inter-molecular interactions with cubic spline functions on a uniformly spaced grid. We defined these potential functions by smoothing and scaling potential functions that were previously calculated for a 1-site CG model of acetic acid.[8]

In order to simplify the presentation, we did not employ distinct functions for all 53 interaction potentials. In particular, we defined the 4 distinct bond potentials and the 5 distinct angle potentials with the same harmonic functions of the bond and angle, respectively. Additionally, we defined all 10 distinct pair potentials with the same function.

We adopted similar simplifications for defining the LD and SG potentials. While this model treats 16 distinct types of local density, we adopted the same Lucy weighting function with $r_{\mathrm{c}} = 0.65$ nm to define the local density, $\rho_{\tau|I}$, of τ type sites around each site, I. The LD potentials, $U_{\rho\tau|\tau_I}$, for site I depend upon the type, τ_I, of site I but not the type, τ, of the

surrounding particles. Similarly, the SG coefficients, $U_{\boldsymbol{\nabla}\tau|\tau_I}$, for site I depend upon τ_I but not τ. Furthermore, the LD potential and SG coefficient functions for different site types are related by scalar multiples.

An important technical detail is that we defined the LD and SG potentials such that their support was within the range of local densities sampled by the LAMMPS simulation. If the range of sampled local densities does not completely cover the support of the LD and SG potentials, then the boundary conditions of the spline basis functions employed in the variational force-matching calculation are not consistent with the simulated LD and SG potentials at the boundary of the sampled local densities, i.e., the basis functions vanish at this boundary but the simulated potentials do not. This not only results in slight errors at the boundaries of the calculated LD and SG functions, but also generates oscillations in the calculated pair forces at r_{c}. Essentially the discrepancy at the boundaries of the LD and SG functions couple to the boundary of the LD contribution to the pair force. Nevertheless, the calculated total potential accurately matches the simulated net forces, i.e., $\chi^2 \approx 0$. Consequently, we suspect that these considerations are not important in the case of systematic coarse-graining because they likely introduce little error in reproducing the true mean forces.

We generated the initial configuration for this third test by employing the LAMMPS `create_atoms` command with a template to randomly place the molecules in a cubic box with sides of length $L = 4$ nm. After performing a brief energy minimization, we simulated the system for 22 ns with the constant nvt fix, while employing a 1 fs time step to integrate the equations of motion for a Nosé-Hoover chain[13–15] with the default chain length, $n = 3$, and a damping time of 100 fs. The simulations employed a 3.0 nm neighbor list, which was checked at every time step. We sampled every 500th configuration from this simulation.

The variational force-matching calculation simultaneously determined 25,109 potential parameters. This calculation employed the same 5,957 spline basis functions that were used to define the non-bonded potentials. This calculation also employed a total of 19,152 spline

basis functions to represent the bonded potentials. We tailored the knot spacings and polynomial degrees to accurately describe each bonded potential. The net forces do not determine unique LD potentials. Rather the non-bonded potentials obtained from force-matching are members of a family of equivalent potentials that generate identical net forces.[6] In the following, we report the member of this family that most nearly matches the simulated potentials. We identify this member based upon an automated procedure previously described in Refs. 18 and 19.

3 Hyperparameters for test calculations

BOCS employs several hyperparameters when performing variational force-matching. As described in the main text, in addition to an input high resolution trajectory, the user must specify the type of interaction potentials, $U_\zeta(x)$, that are to be included in the approximate CG potential, $U(\mathbf{R})$. (The same considerations also apply to the SG coefficient function, $U_{\boldsymbol{\nabla}}$, but we describe interaction potentials for simplicity.) Each interaction potential is a function of a mechanical degree of freedom, ψ_ζ, which may be, e.g., an angle or a type of local density. The user must specify the domain, $[x_{\zeta;\mathrm{lo}}, x_{\zeta;\mathrm{hi}}]$, over which the corresponding force function, $F_\zeta(x)$, should be determined. The lower, $x_{\zeta;\mathrm{lo}}$, and upper, $x_{\zeta;\mathrm{hi}}$, bounds of this domain are typically specified by the minimum and maximum values, respectively, of ψ_ζ that are sampled by the input high resolution trajectory. The user must also specify the type of basis functions (e.g., linear or cubic spline functions) employed to represent $F_\zeta(x)$, as well as the width, $\mathrm{d}x_\zeta$, of the support, $I_{\zeta d}$, for each basis function, $f_{\zeta d}(x)$. In addition to these hyperparameters that define the basis functions for the approximate CG potential, BOCS also employs two additional hyperparameters, ϵ_{trim} and ϵ_{SVD}, to determine the coefficients for these basis functions.

The BOCS ϵ_{trim} parameter determines a threshold for eliminating basis functions that have not been adequately characterized by the input high resolution trajectory. Let $\Delta x_\zeta =$

$x_{\zeta;\text{hi}} - x_{\zeta;\text{lo}}$ be the width of the domain for the $F_\zeta(x)$ force function. The number of basis functions representing $F_\zeta(x)$ is then $N_\zeta = \Delta x_\zeta / \mathrm{d}x_\zeta$. When building the $G_{DD'}$ matrix and b_D vector for the force-matching calculation, BOCS also determines a histogram, $h_{\zeta d}$, that specifies the number of times the ψ_ζ degree of freedom samples the interval, $I_{\zeta d}$, associated with basis function $f_{\zeta d}(x)$. Let $H_\zeta = \sum_d h_{\zeta d}$ be the total number of times that the histogram is updated for the ζ interaction. If $h_{\zeta d} < \epsilon_{\text{trim}} H_\zeta / N_\zeta$, then BOCS eliminates the force field basis vector, $\boldsymbol{\mathcal{G}}_{\zeta d}$, associated with $f_{\zeta d}(x)$ from the variational force-matching calculation, i.e., it trims the corresponding elements from the G matrix and b vector.

BOCS determines the optimal potential parameters, ϕ_*, by solving the normal system of force-matching equations, $G\phi_* = b$, with the trimmed G matrix and b vector. Let the eigenvalues of the trimmed G matrix be ordered $\lambda_1 \geq \lambda_2 \geq \cdots \geq N_D$ and let $\mathbf{v}_1, \mathbf{v}_2, \ldots, \mathbf{v}_{N_D}$ indicate the corresponding eigenvectors. Then $G^{-1} = \sum_{i=1}^{N_D} \mathbf{v}_i \lambda_i^{-1} \mathbf{v}_i^\dagger$. BOCS eliminates from this sum any terms associated with eigenvalues, λ_i, for which λ_i/λ_1 falls below a user-specified threshold, ϵ_{SVD}.

The manuscript reports the results of three tests that demonstrate the accuracy of the LD and SG potentials that we have implemented into BOCS version 5.0 and the PKG-BOCS package in LAMMPS. Below we report the hyperparameters employed in the variational force-matching calculations for each test. We report parameters in BOCS units, i.e., distances are reported in nm, densities are reported in nm^{-3}, and angles are reported in degrees. When parameterizing CG models, we typically employ default values for these hyperparameters $\epsilon_{\text{trim}} = 10^{-3}$ and $\epsilon_{\text{SVD}} = 10^{-6}$. However, in the reported test cases, we sometimes employed smaller values for these hyperparameters in order to more precisely recover the simulated force functions.

3.1 Test 1: 400 particles with linear LD potential

Section 4.1.1 of the manuscript reports the results of force-matching calculations for a system of 400 particles that interact via a linear LD potential. We simulated 5 variants of this

system in which the local density was defined by the 5 weighting functions defined in section 1 above. We performed two independent force-matching calculations for each of the 5 simulated variants.

1. The first force-matching calculation represented U with LD potentials. This calculation employed a trimming parameter $\epsilon_{\text{trim}} = 10^{-10}$ and SVD cutoff $\epsilon_{\text{SVD}} = 10^{-6}$. In the case of the variant employing the Lucy weighting function, we employed a relatively coarse grid spacing, dρ, and selected ρ_{lo} to be somewhat larger than the minimum sampled local density in order to address sparse sampling of configurations with very low local density. Table S2 reports the hyperparameters that define the basis functions for this force-matching calculation.

Table S2: Hyperparameters describing the force-matching calculations in section 4.1.1 that defined U with LD potentials. The first column indicates the weighting function employed to define the local density for both the input simulation and the force-matching calculation. The second column indicates the support specified for the calculated LD force function, $F_\rho(\rho)$. The third and fourth columns indicate the grid spacing and the order of the spline for the basis functions that represented $F_\rho(\rho)$.

w	$[\rho_{\text{lo}}, \rho_{\text{hi}}]$	dρ	Spline Order
DPD	[2.3873241, 946.677]	20	4
Lucy	[100, 832.2593284]	30	4
Shell	[0.6266726, 250.2556252]	20	4
smooth	[1.3369015, 533.9427756]	20	4
sphere	[1.9098593, 758.2039449]	20	4

2. The second force-matching calculation represented U with pair potentials. This calculation employed the same hyperparameters for each of the 5 variants. In particular, it employed a trimming parameter $\epsilon_{\text{trim}} = 10^{-9}$ and SVD cutoff $\epsilon_{\text{SVD}} = 10^{-6}$. Table S3 presents the hyperparameters defining the basis functions that represented the calculated pair force, $F_2(r)$.

Table S3: Hyperparameters describing the force-matching calculations in section 4.1.1 that defined U with pair potentials. The first column indicates that the same parameters were employed for all 5 variants. The second column indicates the support specified for the calculated pair force function, $F_2(r)$. The third and fourth columns indicate the grid spacing and the order of the spline for the basis functions that represented $F_2(r)$.

w	$[r_{\text{lo}}, r_{\text{hi}}]$	dr	Spline Order
*	[0.001, 1.5]	0.01	4

3.2 Test 2: Two particles with LD or SG potentials

Section 4.1.2 of the manuscript reports the results of force-matching calculations for a system of 2 particles that interacted via either LD or SG potentials.

LD potential We performed 5 simulations of 2 particles that interacted with LD potentials. The five simulations corresponded to defining the local density with the 5 weighting functions defined in section 1 above. We performed two independent force-matching calculations for each of the 5 simulations.

1. The first force-matching calculation represented the potential with LD potentials. This calculation employed a trimming parameter $\epsilon_{\text{trim}} = 10^{-11}$ and SVD cutoff $\epsilon_{\text{SVD}} = 10^{-11}$. Table S4 reports the remaining hyperparameters that define the basis functions for this force-matching calculation.

Table S4: Hyperparameters describing the force-matching calculations in section 4.1.2 that defined U with LD potentials. The first column indicates the weighting function employed to define the local density for both the input simulation and the force-matching calculation. The second column indicates the support specified for the calculated LD force function, $F_\rho(\rho)$. The third and fourth columns indicate the grid spacing and the order of the spline for the basis functions that represented $F_\rho(\rho)$.

w	$[\rho_{\text{lo}}, \rho_{\text{hi}}]$	dρ	Spline Order
DPD	[0.00, 1376.6822386]	138	4
Lucy	[0.00, 1208.8031735]	121	4
Shell	[0.00, 362.6577722]	37	4
smooth	[0.00, 773.669330]	78	4
sphere	[0.00, 1103.0995341]	111	4

2. The second force-matching calculation represented U with pair potentials. This calculation employed the same hyperparameters for each of the 5 variants. In particular, it employed a trimming parameter $\epsilon_{\text{trim}} = 10^{-13}$ and SVD cutoff $\epsilon_{\text{SVD}} = 10^{-6}$ for each variant. Table S5 presents the hyperparameters defining the basis functions that represented the calculated pair force, $F_2(r)$.

Table S5: Hyperparameters describing the force-matching calculations in section 4.1.2 that defined U with pair potentials. The first column indicates that the same parameters were employed for all 5 variants. The second column indicates the support specified for the calculated pair force function, $F_2(r)$. The third and fourth columns indicate the grid spacing and the order of the spline for the basis functions that represented $F_2(r)$.

w	$[r_{\text{lo}}, r_{\text{hi}}]$	dr	Spline Order
All indicators	[0.0000, 0.2]	0.0001	2

Square Gradient Case We performed 5 simulations of 2 particles that interacted via SG potentials. The five simulations corresponded to defining the local density with the 5 weighting functions defined in section 1 above. We performed two independent force-matching calculations for each of the 5 simulations.

1. The first force-matching calculation represented the potential with SG potentials. This calculation employed a trimming parameter $\epsilon_{\text{trim}} = 10^{-11}$ and SVD cutoff $\epsilon_{\text{SVD}} = 10^{-11}$. Table S6 reports the remaining hyperparameters that define the basis functions for this force-matching calculation. We represented the SG coefficient function for each variant with a single linear spline function.

2. The second force-matching calculation represented U with pair potentials. This calculation employed a trimming parameter $\epsilon_{\text{trim}} = 10^{-13}$ and SVD cutoff $\epsilon_{\text{SVD}} = 10^{-6}$ for each variant. Table S7 presents the hyperparameters defining the basis functions that represented the calculated pair force, $F_2(r)$.

Table S6: Hyperparameters describing the force-matching calculations in section 4.1.2 that defined U with SG potentials. The first column indicates the weighting function employed to define the local density for both the input simulation and the force-matching calculation. The second column indicates the support specified for the calculated SG coefficient function, $U_{\nabla}(\rho)$. The third and fourth columns indicate the grid spacing and the order of the spline for the basis functions that represented $U_{\nabla}(\rho)$.

w	$[\rho_{\mathrm{lo}}, \rho_{\mathrm{hi}}]$	$\mathrm{d}\rho$	Spline Order
DPD	[0.00, 298.3718880]	298.3718880	2
Lucy	[0.00, 259.6008068]	259.6008068	2
Shell	[0.00, 78.3340522]	78.3340522	2
smooth	[0.00, 167.0996250]	167.0996250	2
sphere	[0.00, 237.8856639]	237.8856639	2

Table S7: Hyperparameters describing the force-matching calculations in section 4.1.2 that defined U with pair potentials. The first column indicates the weighting function employed to define the local density for both the input simulation and the force-matching calculation. The second column indicates the support specified for the calculated pair force function, $F_2(r)$. The third and fourth columns indicate the grid spacing and the order of the spline for the basis functions that represented $F_2(r)$.

w	$[r_{\mathrm{lo}}, r_{\mathrm{hi}}]$	$\mathrm{d}r$	Spline Order
DPD	[0.0000, 0.2]	0.0005	4
Lucy	[0.0000, 0.2]	0.002	4
Shell	[0.0000, 0.2]	0.0005	4
smooth	[0.0000, 0.2]	0.0005	4
sphere	[0.0000, 0.2]	0.0005	4

3.3 Test 3: 125 molecules with LD and SG potentials

Section 4.1.3 of the manuscript reports a force-matching calculation for a system of 125 molecules that interacted with bonded potentials, as well as pair, LD, and SG potentials. The force-matching calculation determined the force function, F_{ζ}, for all interactions except the SG potential. In the case of the SG potential, the force-matching calculation determined the SG coefficient functions, $U_{\nabla \tau_2|\tau_1}$. Table S8 reports the hyperparameters that defined the basis functions for this calculation. The force-matching calculation employed the default trimming and SVD parameters, $\epsilon_{\mathrm{trim}} = 10^{-3}$ and $\epsilon_{\mathrm{SVD}} = 10^{-6}$.

Table S8: Hyperparameters defining the basis functions for the force-matching calculation reported in section 4.1.3. The first column indicates the type of interaction, while the second column indicates the corresponding mechanical degree of freedom. The third column indicates the support specified for the calculated force function, $F_\zeta(x)$, or SG coefficient function, $U_{\boldsymbol{\nabla}\tau_2|\tau_1}(\rho)$. The fourth and fifth columns indicate the grid spacing and the order of the spline for the basis functions that represented each of these functions.

Interaction	ψ_ζ	$[x_{\zeta;\mathrm{lo}}, x_{\zeta;\mathrm{hi}}]$	dx	spline order
All nonbonded pair	r	[0.00, 1.5]	0.01	4
All X|A LD	ρ	[0.00, 6.0]	0.01	4
X|B, X|C, X|D LD	ρ	[0.00, 6.0]	0.1	4
All SG	ρ	[0.00, 6.0]	0.1	4
All Bonds	r_b	[0.00,0.45]	0.0001	2
Angles ABC, BCD, ABA, DCD	ϕ	[0.00, 180]	1	2
Angle CDA	ϕ	[0.00, 180]	1	4
All Dihedrals	ψ	[-180, 180]	3	4

4 Additional analysis of test 3 simulation

Section 4.1.3 of the manuscript reports a simulation and force-matching calculation for a system of 125 molecules that interacted with bonded potentials, as well as pair, LD, and SG potentials. These molecules consisted of 4 types of sites τ = A, B, C, and D, with B and C sites at the interior of the molecule, while A and D sites occur at the surface. Figure 6 of the manuscript reports the simulated force functions and SG coefficient functions. Importantly, all site pairs interact via the same pair potential, while the LD and SG potentials acting on each site, I, are specified by the type, τ_I, of this site. The LD and SG potentials become stronger in the order A $<$ B $<$ C $<$ D, i.e, they are weakest for sites of type A and strongest for sites of type D. Moreover, the LD and SG potentials only generate finite forces when the corresponding local density falls in the range 1.63 nm^{-3}$< \rho_{\mathrm{L}} <$ 5.35 nm^{-3} and 1.91 nm^{-3}$< \rho_{\mathrm{L}} <$ 5.44 nm^{-3}, respectively.

Figure S1 further characterizes the morphology generated by simulations with this model. Panels a and b, which are reproduced from Fig. 7 of the manuscript, illustrate the initial condition of this simulation and the structure generated after 6 ns of simulation. Panels c–f report the distribution of each type of local density, $\rho_{\tau|I}$, around each type of CG site. As

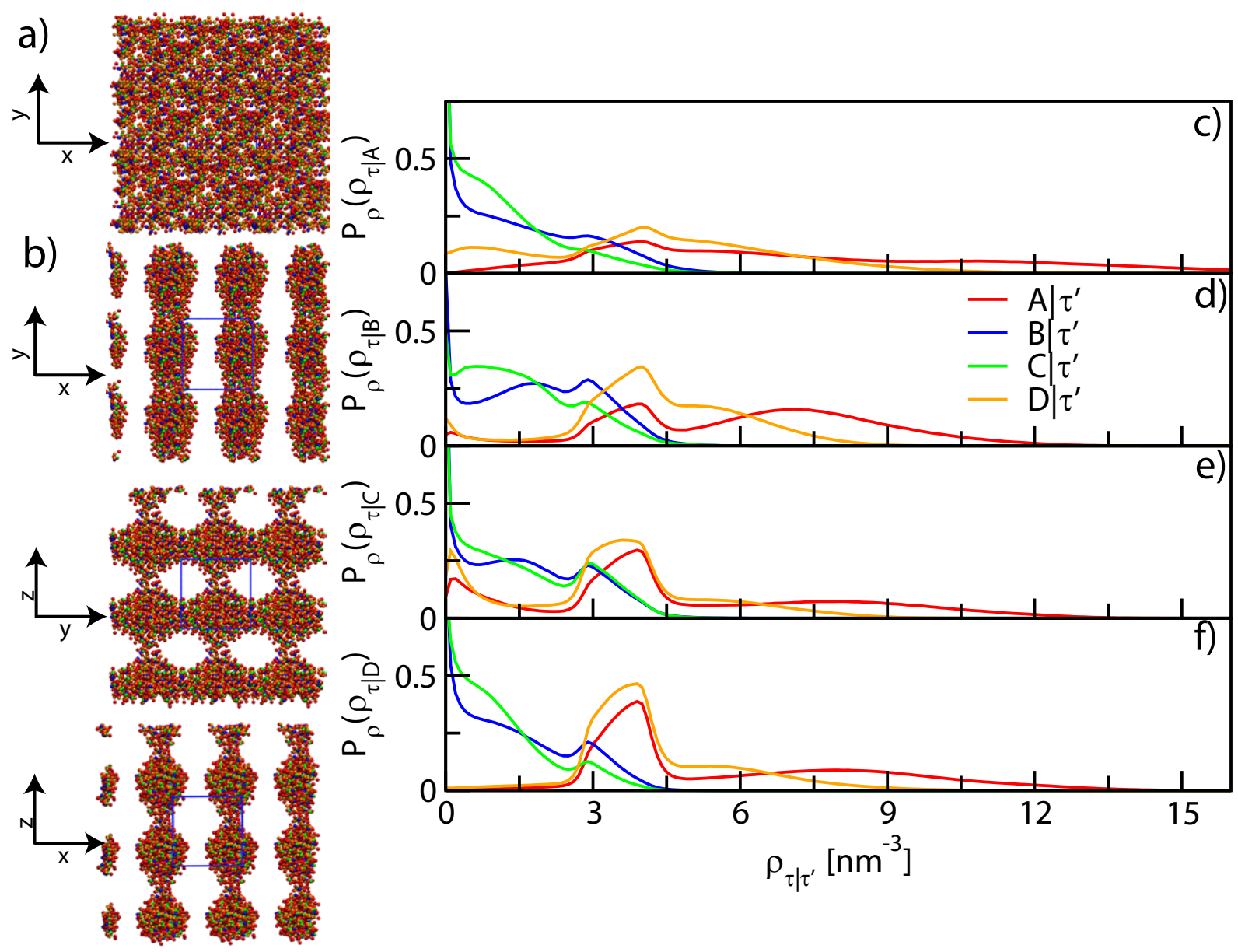

Figure S1: Further analysis of the simulation from section 4.1.3 of the manuscript. Panels and b report orthographic projections of the system at $t = 0$ ns and $t = 6$ ns, respectively. Panels c–f report the simulated local density distributions as a function of $\rho_{\tau|I}$. Panels c, d, e, and f correspond to sites I with $\tau' = \tau_I =$ A, B, C, and D, respectively. In each of these panels, red, blue, green, and orange curves correspond to $\tau =$ A, B, C, and D, respectively.

noted in the main text, the local densities of B and C sites, $\rho_{B,C|I}$ rarely exceed 4.5 nm^{-3}. Additionally, we note that the peak in the LD distributions of A and D sites become increasingly sharp around a site, I, as the type, τ_I, of site I varies in the order order A $<$ B $<$ C $<$ D, which corresponds to the order of the LD and SG potentials.

Figure S2 reports time traces for various contributions to the total nonbonded potential. Panels a and b report the contributions from LD and SG potentials. In particular, we decompose the total LD and SG potentials into contributions from each type of local density around all of the particles, e.g., the A|X LD contribution, $U_{\rho\mathrm{A|X}}$, is defined as the sum of contributions from A local densities around all $I = 1, \ldots, N$ particles, i.e., $U_{\rho\mathrm{A|X}} = \sum_{I=1}^{N} U_{\rho A|\tau_I}(\rho_{A|I})$. Panel c reports the contributions from each nonbonded pair potential. The time traces demonstrate that variations in the pair potentials and, in particular, the AD pair potential dominate the change in the total nonbonded potential during the simulation.

We investigated the influence of periodic boundary conditions upon the simulated mor-

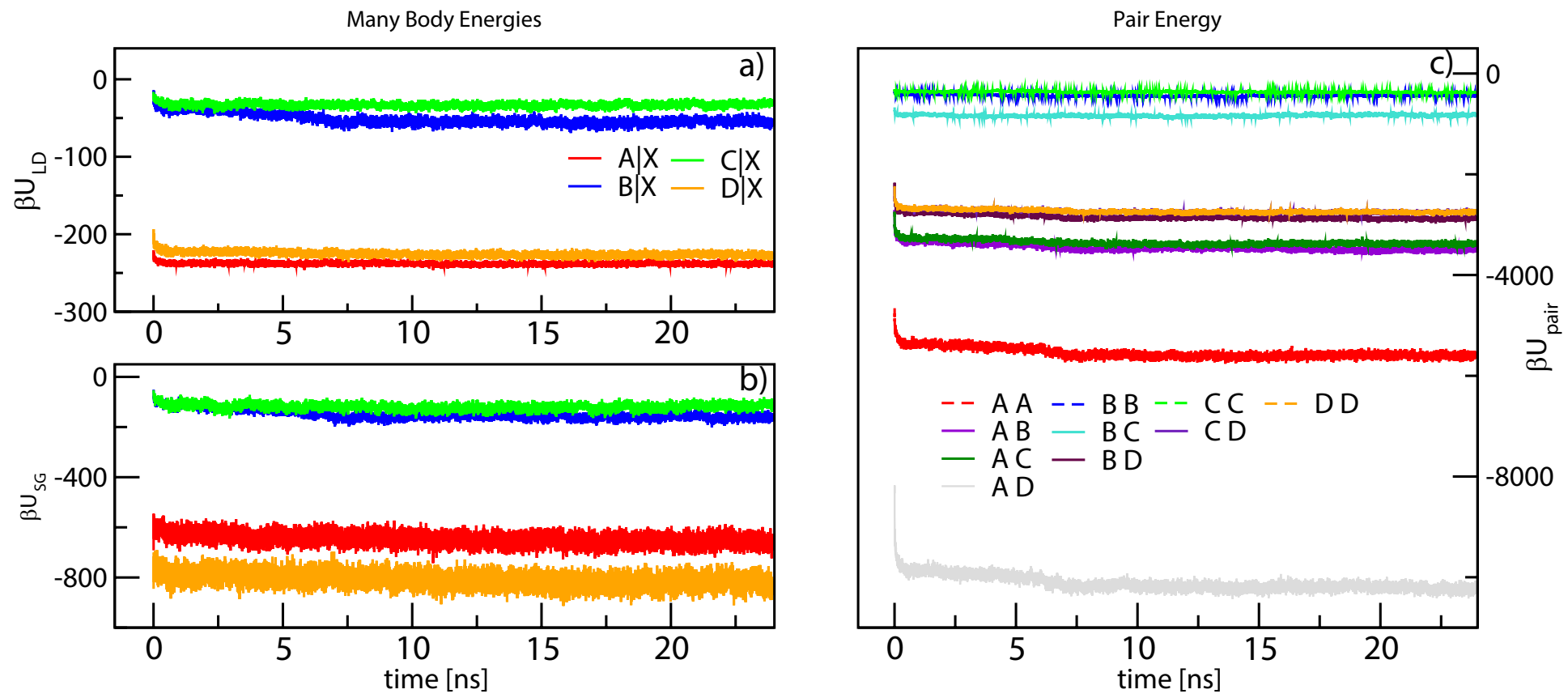

Figure S2: Analysis of the time evolution of the total nonbonded potential. Panels a, b, and c report contributions from LD, SG, and pair potentials, respectively. The LD and SG potentials are decomposed into contributions from each site type, τ, by summing the contributions of this type to the LD and SG potentials for all of the particles.

phology by simulating the same system in a larger simulation box. Specifically, we followed the same protocols described in section 2.3 of this SI, except that we created the initial condition by randomly inserting the 125 molecules into a cubic region with sides of length $L = 4$ nm in the center of a larger periodic cube with sides of length $L = 10$ nm. In this case the system rapidly evolved to form an approximately spherical drop. Figure S3 analyzes this simulation in analogy to Fig. 8 of the manuscript.

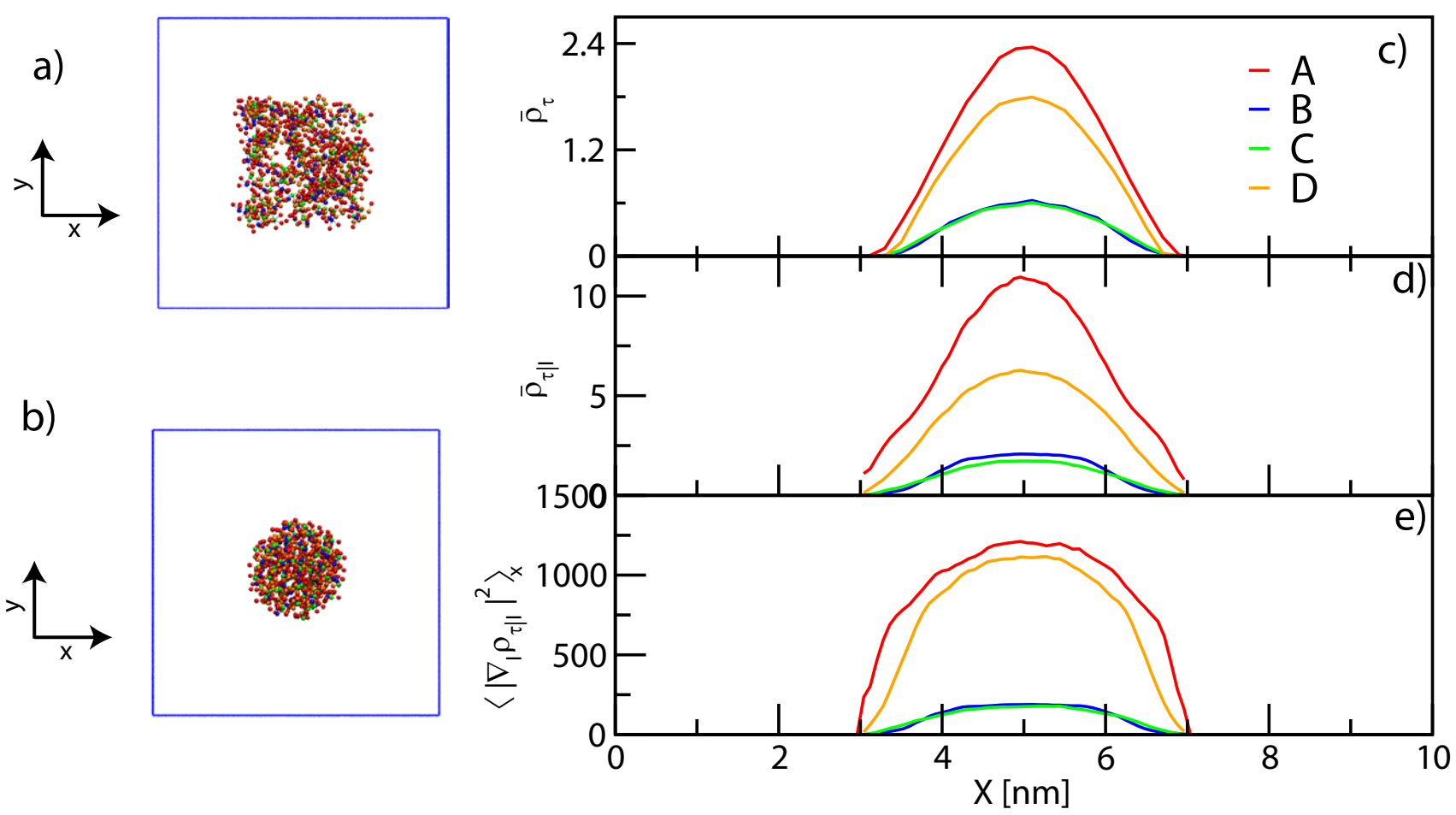

Figure S3: Analysis of simulated morphology. Panel a presents a visualization of the initial configuration, while panel b presents a visualization of the configuration after 6 ns of simulation. Panels c – e characterize the average density of τ = A, B, C, and D sites as a function of the x-coordinate in the simulation box. Specifically, panel c presents the average total density, $\overline{\rho}_{\tau}(x)$, of τ sites; panel d presents the average local density, $\overline{\rho}_{\tau|I}(x)$, of τ sites around each site, I, of type A; and panel e presents the average square gradient of the τ local density, $\left\langle|\boldsymbol{\nabla}_I\rho_{\tau|I}|^2\right\rangle_x$, around each site, I, of type A. Panels c, d, and e report densities and square gradients in units of c) nm^{-3}; d) nm^{-3}; and e)nm^{-8}.

5 Data Availability

The data and analysis for this study[20] are available at the Penn State Data Commons repository[21] repository with doi:10.26208/PW48-SA96.

References

(1) Groot, R. D.; Warren, P. B. Dissipative particle dynamics: Bridging the gap between atomistic and mesoscopic simulation. J. Chem. Phys. **1997**, 107, 4423–4435.

(2) Pagonabarraga, I.; Frenkel, D. Dissipative particle dynamics for interacting systems. J. Chem. Phys. **2001**, 115, 5015–5026.

(3) Warren, P. B. Vapor-liquid coexistence in many-body dissipative particle dynamics. Phys. Rev. E **2003**, 68, 066702.

(4) Moore, J. D.; Barnes, B. C.; Izvekov, S.; Lísal, M.; Sellers, M. S.; Taylor, D. E.; Brennan, J. K. A coarse-grain force field for RDX: Density dependent and energy conserving. J. Chem. Phys. **2016**, 144, 104501.

(5) Wagner, J. W.; Dannenhoffer-Lafage, T.; Jin, J.; Voth, G. A. Extending the range and physical accuracy of coarse-grained models: Order parameter dependent interactions. J. Chem. Phys. **2017**, 147, 044113.

(6) DeLyser, M. R.; Noid, W. Analysis of local density potentials. J. Chem. Phys. **2019**, 151, 224106.

(7) DeLyser, M.; Noid, W. Bottom-up coarse-grained models for external fields and interfaces. J. Chem. Phys. **2020**, 153, 224103.

(8) DeLyser, M.; Noid, W. Coarse-grained models for local density gradients. J. Chem. Phys. **2022**, 156, 034106.

(9) Lucy, L. B. A numerical approach to the testing of the fission hypothesis. Astro. J. **1977**, 82, 1013–24.

(10) Monaghan, J. J. Smoothed particle hydrodynamics. Reports on Progress in Physics **2005**, 68, 1703–1759.

(11) Sanyal, T.; Shell, M. S. Coarse-grained models using local-density potentials optimized with the relative entropy: Application to implicit solvation. J. Chem. Phys. **2016**, 145, 034109.

(12) Sanyal, T.; Shell, M. S. Transferable Coarse-Grained Models of Liquid-Liquid Equilibrium Using Local Density Potentials Optimized with the Relative Entropy. J. Phys. Chem. B **2018**, 122, 5678–5693.

(13) Nose, S. A molecular dynamics method for simulations in the canonical ensemble. Mol. Phys. **1984**, 52, 255–68.

(14) Hoover, W. G. Canonical dynamics: Equilibrium phase-space distributions. Phys. Rev. A **1985**, 31, 1695–7.

(15) Martyna, G. J.; Klein, M. L.; Tuckerman, M. Nosé–Hoover chains: The canonical ensemble via continuous dynamics. J. Chem. Phys. **1992**, 97, 2635–2643.

(16) Dünweg, B.; Paul, W. Brownian dynamics simulations without Gaussian random numbers. International Journal of Modern Physics C **1991**, 2, 817–827.

(17) Brünger, A.; Brooks III, C. L.; Karplus, M. Stochastic boundary conditions for molecular dynamics simulations of ST2 water. Chemical physics letters **1984**, 105, 495–500.

(18) Szukalo, R. J.; Noid, W. A temperature-dependent length-scale for transferable local density potentials. J. Chem. Phys. **2023**, 159.

(19) Lesniewski, M. C.; Remsing, R. C.; Noid, W. G. Revisiting what we lose by coarse-graining: Modeling cooperative hydrophobic phenomena with short-ranged, pair-additive forces. J. Chem. Phys. **2026**, 164, 204111.

(20) Lesniewski, M. C.; DeLyser, M. R.; Noid, W. G. Progress toward a better BOCS: Systematic coarse-graining with local density potentials. 2026; Available from Penn State Data Commons at https://doi.org/10.26208/PW48-SA96.

(21) Penn State Data Commons. Available at http://www.datacommons.psu.edu.
